# Integration of eQTL Analysis and GWAS Highlights Regulation Networks in Cotton under Stress Condition

**DOI:** 10.3390/ijms23147564

**Published:** 2022-07-08

**Authors:** Xiao Han, Chenxu Gao, Lisen Liu, Yihao Zhang, Yuying Jin, Qingdi Yan, Lan Yang, Fuguang Li, Zhaoen Yang

**Affiliations:** 1 State Key Laboratory of Cotton Biology, Chinese Academy of Agricultural Sciences, Anyang 455000, China; hanxiaohanxiao@126.com (X.H.); liulisen2012@163.com (L.L.); zhangyihao0820@163.com (Y.Z.); jinyuying37@126.com (Y.J.); yanqingdi2020@163.com (Q.Y.); yanglan5035@163.com (L.Y.); 2 Zhengzhou Research Base, State Key Laboratory of Cotton Biology, Zhengzhou University, Zhengzhou 450001, China; 202022582017518@gs.zzu.edu.cn

**Keywords:** *Gossypium arbourem*, eQTL, transcriptomes, hotspot, TWAS

## Abstract

The genus *Gossypium* is one of the most economically important crops in the world. Here, we used RNA-seq to quantify gene expression in a collection of *G. arboreum* seedlings and performed eGWAS on 28,382 expressed genes. We identified a total of 30,089 eQTLs in 10,485 genes, of which >90% were trans-regulate target genes. Using luciferase assays, we confirmed that different cis-eQTL haplotypes could affect promoter activity. We found ~6600 genes associated with ~1300 eQTL hotspots. Moreover, hotspot 309 regulates the expression of 325 genes with roles in stem length, fresh weight, seed germination rate, and genes related to cell wall biosynthesis and salt stress. Transcriptome-wide association study (TWAS) identified 19 candidate genes associated with the cotton growth and salt stress response. The variation in gene expression across the population played an essential role in population differentiation. Only a small number of the differentially expressed genes between South China, the Yangtze River region, and the Yellow River region sites were located in different chromosomal regions. The eQTLs found across the duplicated gene pairs showed conservative cis- or trans- regulation and that the expression levels of gene pairs were correlated. This study provides new insights into the evolution of gene expression regulation in cotton, and identifies eQTLs in stress-related genes for use in breeding improved cotton varieties.

## 1. Introduction

Genome-wide association studies (GWAS) are a powerful tool for understanding the genetic basis of trait variation [1]. Genetic correlations by association study between single nucleotide polymorphism (SNP) markers and phenotype, provides candidate regions in the genome. Subsequently, linkage disequilibrium (LD) analysis might gain high-resolution QTL mapping and causal genes. The research over the last decade indicates the genetic basis of a large number of traits for crops [2,3,4]. Quantitative changes in gene expression and protein abundance can be regulated by multiple genetic loci to control complex traits such as plant growth and development. The fast development of next-generation sequencing (NGS) technologies has permitted the analysis of genome-wide gene expression across populations for identification of expression QTLs (eQTLs) [5]. eQTL has also been applied in plants, where it has been used to understand the gene expression network controlling agronomic traits in economically important crops including maize, rice, tomato, and cotton [6,7,8,9]. For cotton, eQTL maps uncover the critical roles of genomic variation in the regulation and regulatory network for fiber and high temperature stress [10,11]. In addition, eQTLs can be combined with phenotypic traits to perform transcriptome-wide association studies (TWAS) to identify critical genes’ associated traits [12]. TWAS has been successfully applied in cotton fiber and heat stress to identify critical genes for important traits [10,11].

The growth and resistance of cotton seedlings provide the foundation for high and stable cotton yield. Several studies have elucidated cotton growth and abiotic stress related genes, but the genetic architecture of the regulation network is much less well understood. *Gossypium arboreum* (A_2_) originated from a common ancestor of the A genome of *G. hirsutum* [13], initially introduced into China >1000 years ago, and possesses several stress tolerance traits, including pest resistance and salt tolerance [14,15,16]. To identify the genetic loci contributing to these traits, we performed mRNA-seq on a previously reported core collection of diploid *G. arboreum* accessions and obtained gene expression profiles of seedlings. We then performed eGWAS analysis that identified trans- and cis-eQTLs across 13 chromosomes which revealed eQTL hotspots. It was found that genes associated with a given hotspot are functionally associated with each other. Moreover, we used TWAS analysis to integrate the cis-eGene eQTLs with GWAS for salt-tolerance which identified important candidate genes. We compared the differentially expressed genes (DEGs) with genes located in regions with high F*st*, and found that only a small subset of high-F*st* genes overlap the list of DEGs, suggesting that both DEGs and genetic differentiation play essential roles in the geographic structuring of the *G. arboreum* population.

## 2. Results

### 2.1. GWAS Analysis for Gene Expression

Gene expression functions as a bridge between the genotype and the phenotype. To understand the contribution of gene expression in phenotype variation among cotton plants with different genotypes, we performed mRNA-seq analysis of a core collection comprising 214 Chinese *G. arboreum* accessions. In total, we obtained ~7 billion 150-bp paired-end reads (1.9 Tb) with an average of 34.5 million reads per accession (Table S1 and Figure S1a). Among all predicted genes in the genome, a total of 4868 genes were not detected by mRNA-seq and were excluded from the downstream analysis. In addition, we filtered genes that were detected in <20% of the test accessions (Table S2 and Figure S1b–d). In the end, 28,382 genes (69.29%) were retained for further analysis.

We used a total of 1,425,003 high-quality SNPs in the 214 accessions from GWAS analysis. We considered a *p*-value of 1.0 × 10^−5^ (1/n, where n is the effective number of independent SNPs) as the significant threshold for SNP identification. This allowed us to discover a total of 7,978,756 significant SNPs associated with 27,617 genes. We combined significant SNPs under linkage disequilibrium (LD) on a single LD region and considered the most significant SNP (termed lead SNP) as the eQTL. This strategy allowed us to identify a total of 30,089 lead SNPs overlapping 10,485 genes, representing 25.6% of the total number of genes that were regulated by eQTLs.

### 2.2. Identification of Trans- and Cis-eQTLs

**Figure 1:**
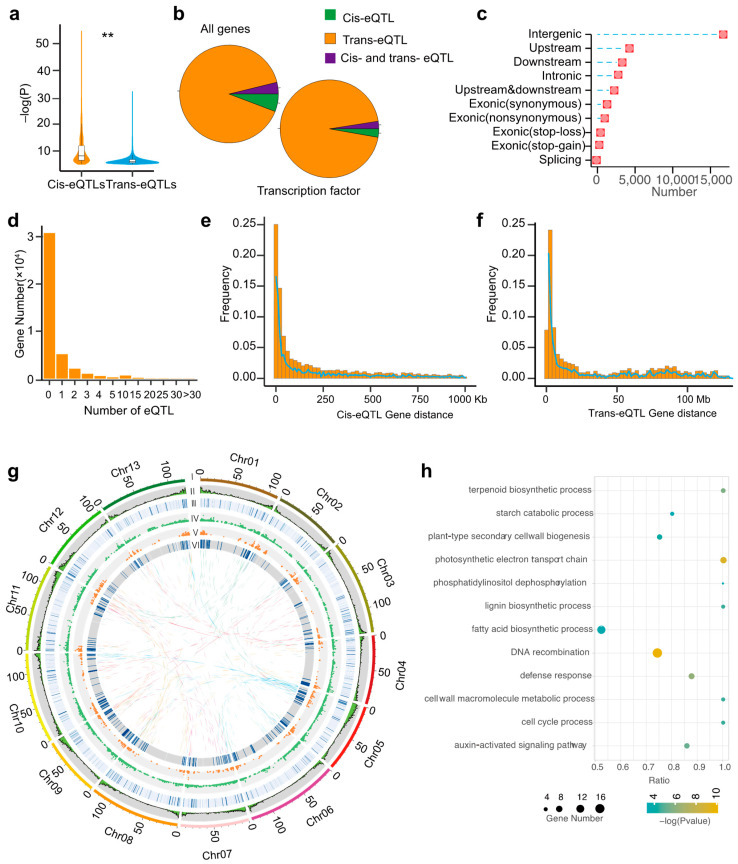
Characterization of eQTLs using RNA-Seq data of seedling samples. (**a**) The comparison of significance between cis-eQTLs and trans-eQTLs. Student‘s test, ** *p* < 1 × 10^3^ (**b**) The proportion of eQTLs that are cis-eQTLs, trans-eQTLs, or are both cis-eQTLs and trans-eQTLs in the categories of all genes or transcription factors (**c**) Number of eQTLs belonging to each functional category. (**d**) Distribution of the number of eQTLs for genes which were regulated by eQTLs. (**e**) Distribution of the distance between cis-eQTLs and the genes they regulate. (**f**) Distribution of distance between trans-eQTLs and the genes they regulate. (**g**) Integration of eQTLs and hotspots. I—the thirteen chromosomes of *Gossypium arboreum*. II—gene density in a 1 Mb window. III—heatmap of gene FPKM. IV—trans-eQTLs (*p* < 1 × 10^5^) association plot. V—cis-eQTLs (*p* < 1 × 10^5^) association plot. VI—histogram of hotspots. (**h**) Functional enrichment analysis for eQTL-regulated genes.

Following a previous report, when the distance between the eQTL loci and their target genes is smaller than 1 Mb, the eQTLs are defined as cis-eQTLs [6,17]. The remaining eQTLs are classified as trans-eQTLs, which constitute the majority of eQTLs uncovered in our study (27,262, accounting for 91.8%). The number of cis-eQTLs, which was much lower (2467), accounted for 8.2% of all the eQTLs, but the correlation coefficients between the different SNPs and gene expression levels were significantly higher than those observed for trans-eQTLs (Figure 1a). We also found that transcription factors (TFs), the important transcription trans-regulators, tend to regulate gene expression through trans-eQTLs, as the proportion of these sites was enriched in TFs compared to all eGenes (Figure 1b). We then compared the genomic locations of cis- and trans-eQTLs and found a similar genomic distribution for both eQTL types: both cis- and trans-eQTLs were enriched within gene-dense euchromatin regions where transcription is more active (Figure 1g and Figure S2). Furthermore, we discovered that most of the lead SNPs, including cis-eQTLs and trans-eQTLs, were located in non-coding regions of the genome, mostly in the intergenic areas, followed by the regions upstream and downstream of the genes and the intronic regions, while a few were found in exonic regions, which had large genetic effects such as nonsynonymous, stop-loss, stop-gain, and splicing mutations (Figure 1c and Figure S3).

We further classified eQTLs and eGenes into three distinct categories based on the distance between them: class I, those located on the same chromosome at a distance <1 Mb; class II, those located on the same chromosome at a distance >1 Mb; and class III, those located on different chromosomes (Figure S4). We then compared the *p*-values between these three classes and found that the −log (*p*) values of class I were the highest, while those of class II were significantly higher than those of class III. These results indicate that distance between eQTLs and their respective target eGenes could be an important factor affecting the strength of the association. In general, each eGene was targeted by an average of 2.8 eQTLs and more than 50% of eGenes were regulated by a single eQTL (Figure 1d). The average distance between cis-eQTLs and target eGenes was ~50 kb. In contrast, the distance between trans-eQTL and target eGenes on the same chromosome was ~50 Mb (Figure 1e–f). Additionally, to identify the potential biological functions of the eQTL target genes, we performed a gene functional enrichment analysis. We found that the target genes for eQTLs were enriched (*p*-value < 0.05) in genes highly associated with plant resistance and growth (Figure 1h).

### 2.3. Validation of Cis-eQTLs Using a Luciferase Assay

**Figure 2:**
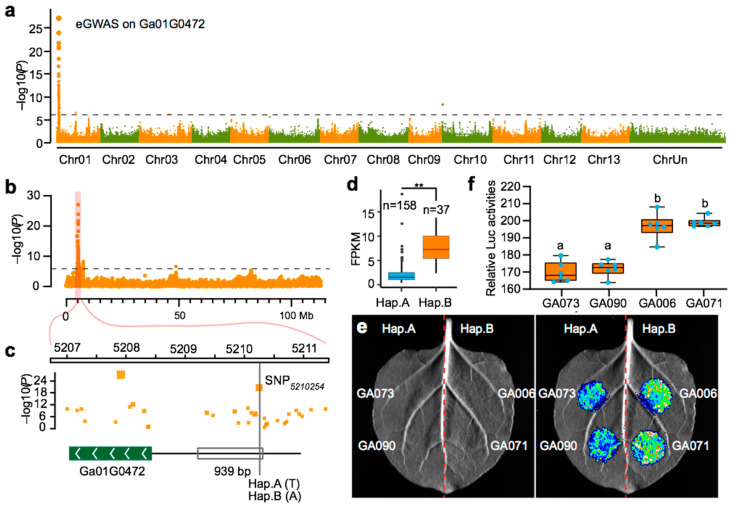
Cis-eQTL regulated gene expression. (**a**) Genome-wide association study (GWAS) on *Ga01G0472* expression. (**b**) Manhattan plot of the eGWAS signal of *Ga01G0472* on chromosome 01. (**c**) The position of the 939-bp fragment used in the *Ga01G0472* promotor used in the Luc assay showing associated SNPs. The lead SNP, Chr 01:5210254, is indicated by a vertical line. (**d**) The divergence of *Ga01G0472* expression between different alleles of the cis-eQTL. Student‘s test, ** *p* < 1 × 10^3^. (**e**) Luminescence of *Nicotiana benthamiana* leaves infiltrated with vectors for containing different alleles of the *Ga01G0472* a promoter driving *LUC* (*Ga01G0472* pro:Luc). Fragments from accessions GA073, GAO90, GA006, GA071 for both Hap. A and Hap. B were used. (**f**) Quantification of the luminescence signals shown in (**e**).

Although a high number of eQTLs have been previously identified, most have not been experimentally validated. The close location of cis-eQTLs to their targeted eGene makes them suitable candidates for validation using a luciferase (luc) activity assay. *Ga01G0472* encodes a NB-ARC family protein and is predicted to be regulated by a cis-eQTL in the region near the telomere on Chr01 (Figure 2a,b). The eQTL is located in the 5207–5211 kb region containing an SNP cluster on the *Ga01G0472* promoter. Within the cluster, SNP_5210254_ was the most significant (*p*-value = 2.681 × 10^−21^) and formed two haplotypes termed Hap. A (T) and Hap. B (A) (Figure 2c). The accessions carrying Hap. B had significantly higher expression levels than those carrying Hap. A (Figure 2d). We next cloned 939-bp segments of the two haplotypes containing the cis-eQTL into the pLL00r vector, designated as GA073 (BaoShanZiHua) and GA090 (AnHuiGuiChiZhongMian) carrying Hap. A, and GA006 and GA071 carrying Hap. B upstream of the luciferase gene. The activities of these segments were analyzed in *Nicotiana tobacum* leaves using *Agrobacterium tumefaciens* infiltration. The vectors containing the segments from the accessions carrying Hap. B (GA006 and GA071) exhibited stronger luminescence than those carrying Hap. A (GA073 and GA090), while no differences in activity were seen between reporters carrying the same haplotype. These results are in accordance with the observed expression differences for *Ga01G0472* in these accessions (Figure 2e,f). This validates the effect of this eQTL and suggests that the newly discovered cis-eQTLs are able to affect promoter activity and regulate gene expression levels.

### 2.4. Trans-eQTLs Hotspot Clusters Are Associated with Seedling Development and Salt Stress

**Figure 3:**
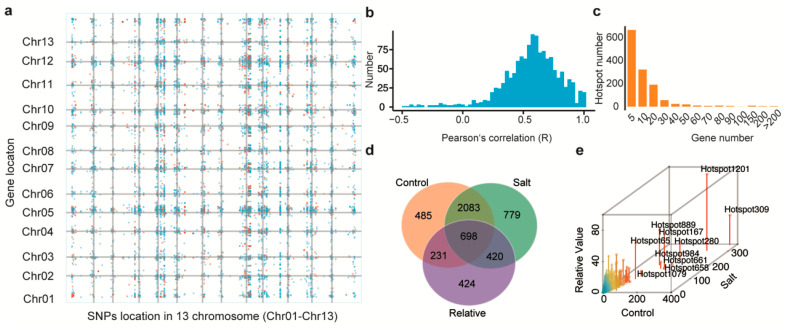
Genomic distribution of trans-eQTLs hotspots. (**a**) Dot plot showing hotspots and their regulated genes on 13 chromosomes. The color of each dot represents the significance (*p*-value) of each eQTL-gene association, with log(*p*-value) > 7 in red and log(*p*-value) < 7 in blue. (**b**) Distribution of R value means for hotspot genes. (**c**) Distribution of gene number of hotspots. (**d**) Venn diagram showing the overlap of genes significant related to the control, salt, and relative categories. (**e**) Top 10 hotspots in genes related with traits for control, salt, and relative value.

Groups of functionally related genes regulated by single eQTLs are called hotspots [18]. We used hot_scan to search potential hotspots in each chromosome of *G. arboreum*. We detected 1298 hotspots (*p*-value < 0.05) associated with 6599 genes across all 13 chromosomes (Figure 3a). We then calculated the correlation between hotspots and associated genes and the results showed positive correlation for most of the genes (Figure 3b). The majority of hotspots (89%) were associated with <20 genes (Figure 3c) and >600 hotspots were only associated with 5 genes.

Cotton growth and fiber yield are affected by seedling development and salt stress. We next focused on analyzing fifteen traits. Those traits were divided into three categories: salt (GR_150_, FW_150_, SL_150_, WC_150_ and EC_150_), control (GR_control_, FW_control_, SL_control_, WC_control_ and EC_control_), and relative (relative GR (RGR), relative FW (RFW), relative SL (RSL), relative WC (RWC), and relative EC (REC)). We calculated the Pearson correlation coefficients between each of these traits and the expression levels of genes, which identified 24,549 genes showing significant correlation. Among them, 7848 genes were associated with eQTLs and 5120 with hotspots. Only 698 hotspot-correlated genes were found to be significantly associated with all three trait categories (Figure 3d). We counted the gene frequency of each hotspot by the three categories, and used it to rank the hotspots. The top ten hotspots were hotspot 309, hotspot 1201, hotspot 280, hotspot 167, hotspot 661, hotspot 889, hotspot 658, hotspot 984, hotspot 65, and hotspot 1079 (Figure 3e). A comparison of the phenotypes between haplotypes using the lead SNP from each hotspot revealed that 6 of the 10 hotspots were significantly associated with at least one of the traits (Figure S5).

### 2.5. Cis-Regulated Genes Are Associated with Stress-Related Traits

TWAS have been successfully used to discover candidate genes for different traits based on the expression of cis-regulated genes [12]. We used this approach to identify genes with potential roles in salinity tolerance. The following traits were analyzed in cotton seedlings under salinity (150 mM NaCl) and normal (control) conditions: seed germination rate (GR), fresh weight (FW), stem length (SL), water content (WC), and electric conductivity (EC). GWAS analysis detected 4160 significant SNPs (*p*-value < 1 × 10^−5^) associated with the above traits (Table S3). As there were no expression weights available for expression imputation, we used 1011 cis-eQTL genes to functionally compute these weights for *G. arboreum*. Next, we performed a TWAS analysis to integrate eQTL and GWAS information. We used five salt tolerance related traits, including seed germination rates (GR), fresh weight (FW), stem length (SL), water content (WC), electric conductivity (EC) under 0 mM(control) and 150 mM (150). The relative value of the traits was calculated as follows: relative value = value under stress treatment/value under control treatment. Hence, our TWAS analysis included 15 phenotypic features: GR_150_, FW_150_, SL_150_, WC_150_, EC_150_, GR_control_, FW_control_, SL_control_, WC_control_, EC_control_, relative GR (RGR), relative FW (RFW), relative SL (RSL), relative WC (RWC), and relative EC (REC). This allowed us to identify 19 transcriptome-wide significant associations, including three for FW_150_, 3 for REC, four for SL_150_, three for WC_control_, and six for WC_150_ (Table 1). Among them, an annexin D2 gene (*Ga11G3524*) encoding a calcium-binding protein was found to be correlated with WC_150_. Overexpression of its tomato homolog AnnSp2 can increase plant drought and salt tolerance through ABA synthesis and ROS elimination [19]. Another gene, *Ga10G0163* encoding coronatine-insensitive protein 1 (COI1) is also associated with WC_150_ tolerance. In Arabidopsis, COI1 is required for the downregulation of NRT1.5 involving the JA signal induced by cadmium or salt [20]. In addition, the IAA14 gene (*Ga03G0409)* encoding an auxin-responsive protein and its Arabidopsis homolog plays a role in abiotic stress response through interaction with Drought-induced 19 (Di19) protein [21,22]. 

We then combined the SNPs identified by GWAS with eQTL loci. To do this we defined eQTL loci with more than three significant GWAS SNPs as QTLs. Using this strategy, we identified a total of 154 QTLs containing 3143 SNPs. For each of these QTLs, we determined if the expression of the gene significantly differed between the two haplotypes of the most significant SNP. This identified 105 QTLs that were significantly associated with traits. For instance, QTL (Chr08:113453649–114064078) is linked to RFW and RSL and 16 eGenes (*Ga01G1789*, *Ga02G0149*, *Ga04G1991*, *Ga08G1871*, *Ga08G1873*, *Ga08G1884*, *Ga08G2627*, *Ga10G0833*, *Ga10G2229*, *Ga11G2943*, *Ga13G1306*, *Ga13G1920*, *Ga13G1949*, *Ga13G2529*, *Ga14G0186*, and *Ga14G1716*) (Figure S6a). *Ga08G1871*, *Ga08G1873*, and *Ga08G1884* were cis-regulated by the QTL. Most of the eGenes are responsive to abiotic and biotic stress (Table S4). The two haplotypes of the most significant SNP for QTL (Chr08:113453649–114064078) exhibited significant difference for RFW, RSL and the expression of *Ga08G1884* (Figure S6b). Based on Pearson’s correlation analysis, eGenes and traits showed complicated relationships (Table S5). Therefore, TWAS and a combination of GWAS and eQTL analysis provided effective ways to uncover the genes and gene networks related to salt stress tolerance.

### 2.6. eQTL Hotspot309 Regulates Cell Wall Biosynthesis and Salt Stress

**Figure 4:**
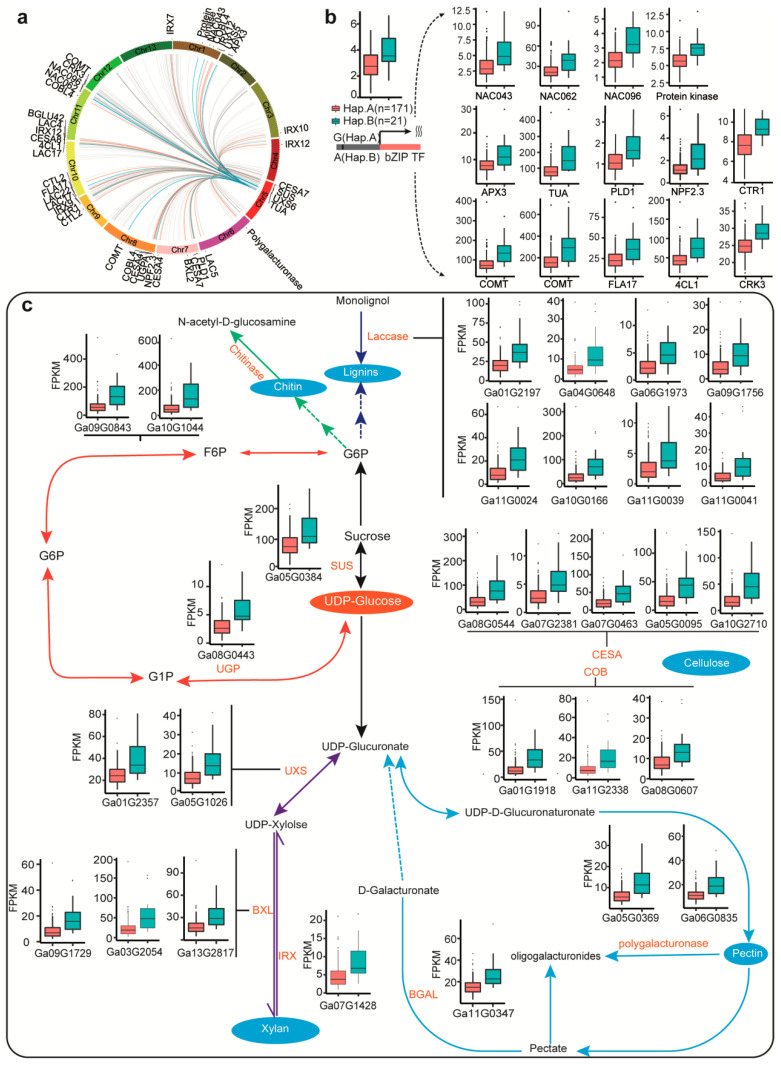
Genes regulated by the trans-eQTLs hotspot 309 are related to cell wall biosynthesis and salt stress. (**a**) The 29 genes involved in cell wall synthesis and 14 genes involved in salt stress were linked to hotspot 309 on Chr05. (**b**) The divergence of the adjacent candidate gene *Ga05G2977* and 14 genes involved in salt stress between different alleles of the lead SNP. (**c**) The divergence of 29 genes involved in cell wall synthesis between different alleles of the lead SNP.

We found that hotspot 309 regulated 325 eGenes that are involved in cell wall biosynthesis and salt stress response as shown by functional enrichment analysis (Tables S6–S8). They are associated with 12 traits (EC_control_, EC_150_, FW_control_, FW_150_, SL_control_, SL_150_, RSL, WC_control_, WC_150_, RWC, GR_150_, RGR) (Figure S7). We further identified their homologues in *Arabidopsis thaliana*, among which 29 have known functions in cell wall biosynthesis and 14 eGene in salt stress response (Table S9, Figure 4a). Network analysis was carried out for the genes orthologous to those eGenes in hotspot 309 using the *Arabidopsis thaliana* STRING database. We found that cell wall biosynthesis-related eGenes and salt stress-related eGenes were distributed in two interconnected networks (Figure S8). The hotspot 309 and most of the eGenes showed a positive correlation. 

The location of hotspot 309 is close to *Ga05G2977*, a basic leucine zipper transcription factor whose ortholog in *Arabidopsis thaliana* is *AT2G40620*, which has a role in cell elongation and network regulation of secondary cell wall synthesis [23]. The expression of *Ga05G2977* significantly differed between the two haplotypes for the lead SNP (Chr05:32126627) of hotspot 309, indicating a close association between *Ga05G2977* and the SNP. The expression of eGenes in hotspot 309 differed significantly between the two haplotypes for the lead SNP. All the cell wall biosynthesis and salt stress related eGenes are different between the two haplotypes of hotspot 309. They were expressed at significantly higher levels in Hap. B than in Hap. A, indicating that they have a synergistic regulatory effect (Figure 4b,c).

To understand whether hotspot 309 is related to salt stress tolerance, we compared the traits of the two haplotypes for the lead SNP. FW_control_ and SL_control_ showed significant differences, higher levels in Hap. B than in Hap. A, was same with eGenes. There was no difference in salt tolerance phenotypes (FW_150,_ RFW, SL_150_ and RSL) between Hap. B and Hap. A. On the contrary, there was no difference GR_control_ and GR_150_ between Hap. B and Hap. A, but RGR showed significant different. Interestingly the levels of Hap. A was significantly higher than Hap. B for RGR. The traits of GR_control_ and GR_150_ was no different between Hap. B and Hap. A (Figure S9). This indicates that the growth traits of Hap. B were better than those of Hap. A under normal conditions. However, the advantage of Hap. B was lost under salt stress. Therefore, we speculated that Hap. B was beneficial for cotton growth but detrimental for salt tolerance. 

### 2.7. Gene Expression Changes Contribute to Population Divergence

**Figure 5:**
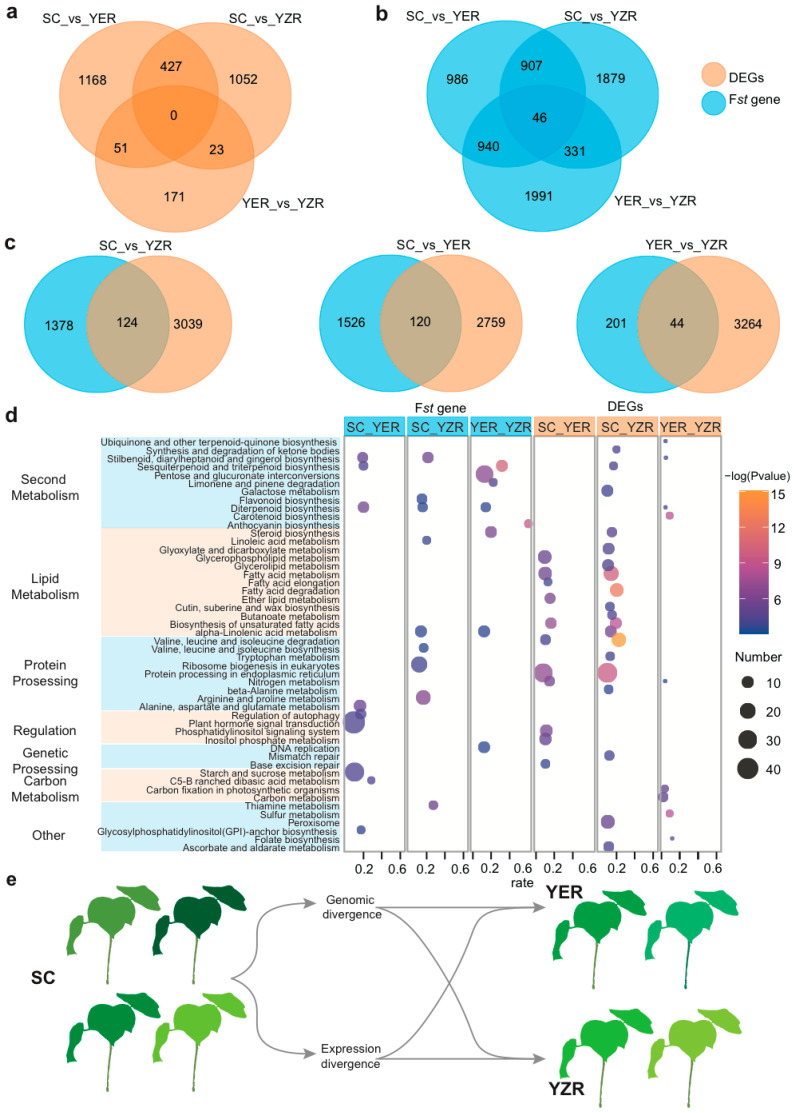
Analysis of different geographical distributions of *G. arboreum.* (**a**) Venn diagram showing the overlap of DEGs between South China (SC), Yellow River region (YER) and Yellow River region (YER). (**b**) Venn diagrams show the overlapping genes with a top 5% of pairwise fixation statistic (F*st*) between SC, YER, and YZR. (**c**) Venn diagram shows the overlapping genes with different expression and top 5% pairwise fixation statistic (F*st*) between SC, YER, and YZR. (**d**) KEGG enrichment of top 5% of F*st* genes and different expression genes between SC, YZR, YER. (**e**) Relationship of geographical distributions of *G. arboretum*.

Our previous study showed that the growth area of *G. arboreum* accessions can be divided into three geographic and ecological groups: South China (SC), the Yangtze River region (YZR), and the Yellow River region (YER) [24,25]. *G. aboreum* was introduced into China from the south; however, it remains unknown whether *G. aboreum* was transmitted from SC to YZR and then to YER, or from SC to YZR and YER at the same time. In order to resolve this question, we first compared 11 agronomic traits from accession lines grown in SC, YZR, and YER as our previous report [23] and found that these traits are more divergent between SC and YZR and between SC and YER than those between YZR and YER. We then randomly selected an equal number (20) of accessions from the SC, YZR, and YER groups to analyze the differentially expressed genes (DEGs) between every two of the three groups (i.e., SC versus YZR, SC versus YER, and YZR versus YER). We repeated the analysis 30 times and filtered DEGs that appeared more than 15 times. The largest number of DEGs came from the comparison between SC and YER (SC vs. YER) (1646) followed by SC vs. YER (1502) and then YZR vs. YER (245), indicating that YZR and YER are weakly differentiated in terms of gene expression (Figure 5a). In addition, our previous study showed that the SC group has the highest nucleotide diversity (π = 0.211 × 10^−3^), followed by YZR (π = 0.197 × 10^−3^), and YER (π = 0.199 × 10^−3^) groups, thus YZR and YER have similar nucleotide diversity [23]. Hence, these data strongly support that the *G. arboreum* accessions of the YZR and the YER groups descend from SC accessions.

We next evaluated whether the above DEGs experienced artificial selection during domestication and migration by comparing top 5% F*st* values between different geographical regions (SC versus YZR, SC versus YER, and YZR versus YER). We found a very small subset of genes (46) that are shared (Figure 5b). Similarly, a very small number of DEGs were shared by the three groups, indicating that each group is specifically differentiated. We then compared the set of genes with high F*st* and DEGs in each group and found little overlap between the two gene sets in all three comparisons (Figure 5c) [23]. Results from KEGG enrichment analysis for the DEGs and F*st* between different geographical regions mainly include seven categories of function: carbon metabolism, genetic processing, lipid metabolism, protein processing, regulation, second metabolism, and others (Figure 5d, Table S11). KEGG of DEGs were correlated with lipid metabolism, protein processing, secondary metabolism, but KEGG of F*st* were mainly correlated with secondary metabolism (Table S10). In summary, our results suggested that *G. arboreum* was initially cultivated in South China before being cultivated in the Yangtze River region and the Yellow River region. The environmental change that accompanied cultivation expansion led to genomic divergence and changes in gene expression (Figure 5e).

### 2.8. eQTL Divergence among Five Models of Gene Duplication

**Figure 6:**
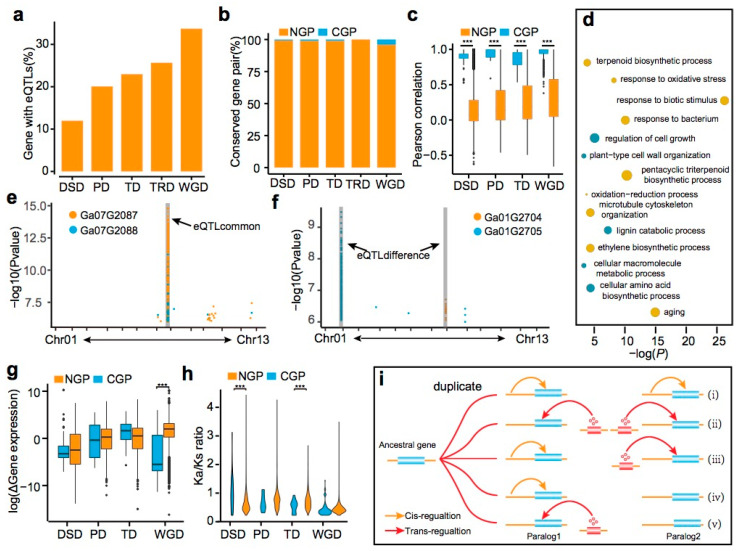
The landscape of eQLTs in five models for gene duplication in *G. arboreum*. (**a**) Percentage of duplicated genes associated with eQTLs. (**b**) Percentage of conserved gene pair for duplicates among five gene duplication models. (**c**) The divergence of the correlation between conserved gene pairs and non-conserved gene pairs across four gene duplication models of duplicate. (**d**) Gene ontology (GO) enrichment of tandem duplication genes. Student‘s test, *** *p* < 1 × 10^4^. (**e**) eQTLs on *Ga07G2087* and *Ga07G2088* expression. (**f**) eQTLs on *Ga01G2704* and *Ga02G2705* expression. (**g**) The divergence of the gene expression between conserved gene pairs and non-conserved gene pairs across four gene duplication models. Student‘s test, *** *p* < 1 × 10^4^. (**h**) The divergence of the Ka/Ks between conserved gene pairs and non-conserved gene pairs among four gene duplication models. Student‘s test, *** *p* < 1 × 10^4^. (**i**) A suggestive model for eQTLs divergence in the duplicated gene pairs.

Gene duplication is a fundamental process affecting genome evolution [26]. There are five different types of duplications based on the location of the duplicated genes, including dispersed duplication (DSD), proximal duplication (PD), tandem duplication (TD), transposed duplication (TRD), and whole-genome duplication (WGD) [27]. Our results show that eQTLs regulated the expression of 33.7% WGDs, 25.7% of TRD, 23.0% TD, 20.1% PD, and 12.0% DSD (Figure 6a). In contrast, the Ks/Ka ratio of duplicate genes showed an inverse pattern, with WGDs-derived genes having the smallest Ks/Ka ratio, followed by TRD, TD, PD, and DSD (Table S11). These results indicate that DSD, TD, and PD that have been preserved at a younger age experienced more rapid sequence and expression regulation divergence than other duplication types.

We further classified these duplicated genes with eQTLs into conserved gene pairs (CGP), which corresponded to those that were regulated by the same eQTLs and non-conserved gene pairs (NGP), representing genes that were regulated by different eQTLs. We found 10%, 8%, 7%, 5%, and 0% CGP in WGD, TDs, PDs, TDR, and DSDs, respectively, as well as a higher correlation within CGP as compared to NGP genes (Figure 6b,c). We examined TD-derived genes as an example to further elucidate the divergence between the eQTLs. The KEGG analysis showed that stress-related pathways such as “response to oxidative stress”, “response to biotic stimulus”, and “response to bacterium” were enriched in TDs (Figure 6d). A total of 727 eQTLs were identified for 752 genes, of which only 25 co-regulated the 50 tandem duplicate genes. For example, *Ga07G2087* and *Ga07G2088*, both encoding MYB family transcription factors, were regulated by the same cis-eQTL on Chr07, whereby the expression levels of these two genes had positive correlation in the population (Figure 6e). In contrast, *Ga01G2704* and *Ga01G2705*, which encode lipid transfer-like proteins, have different eQTLs on Chr01 and Chr07, respectively (Figure 6f). In general, the expression of genes derived from WGD were more conserved in CGPs than in NGPs. However, no significant differences were observed between CGPs and NGPs in other types of duplications (Figure 6g). It is interesting that CGPs have smaller Ks/Ka ratios than NGPs in WGD, but higher in the case of DSD (Figure 6h).

We next analyzed the divergence between eQTLs between four models of gene duplication. In most situations, both members of a duplication pair (8.74%) were regulated by trans-eQTLs (only 0.14% were regulated by cis-eQTLs). Furthermore, we also found cis- and trans-eQTL conversion within two members of a duplicate pair (Figure 6i). For example, *Ga01G0117* is regulated by a trans-eQTL, but its counterpart, *Ga01G0118*, is regulated by a cis-eQTL. These results suggest that trans- or cis-regulation for most of the duplicate gene pairs is conserved during the course of evolution, and that cis- and trans-eQTLs conversion is an important mechanism for the divergence of gene expression levels in duplicated genes.

## 3. Discussion

A large number of genetic variants have been identified through GWAS analysis in cotton populations. However, how these genetic variants contribute to variation in gene expression remains unknown. Here, we demonstrated that eQTL mapping represents a high-throughput approach that can link regulatory variants to gene expression in seedlings. We mapped 30,089 eQTLs regulating 10,485 genes that are enriched for cell wall and stress-related functions. The percentage of cis-eQTLs was lower than most studies in the past, which might be due to improvement of detectability for the cis-eQTLs [28,29,30], the use different software for the analysis, or the strictness of the threshold applied [7,31]. Nevertheless, more regulatory variants could be uncovered if RNA-Seq data from different developmental stages and growth regions were generated. It is important to note that more than half of the eQTLs we uncovered are located in intergenic regions. This observation matches those of earlier studies [28,29,30]. Nevertheless, the mechanisms of how eQTLs contribute to gene regulation and phenotype expression are unclear. Here, we found relative significance decreases with increase in the distance between the eQTLs and the target eGenes, consistent with previous findings [11,32]. This may be caused by the SNP which alters the transcription factor binding site or regulatory element causing effects on gene expression [27,33]. Furthermore, we demonstrated the effect of different haplotypes of the eGWAS on gene expression. We also performed a TWAS analysis to unveil a direct connection between gene expression and phenotype using eQTL and GWAS data, which facilitated the understanding of the regulatory roles of genetic variants in shaping phenotypic differences between accessions.

Salt stress significantly inhibits the growth, development, and productivity of cotton, and cotton plants are more sensitive to salinity during the early seedling growth stage after the germination stage [34,35]. In this study, using eQTL regulating genes and traits-related genes, we detected an association between hotspot 309 and salt stress tolerance. This hotspot is associated with salt tolerance related traits such as stem length, fresh weight, and seed germination rates. The candidate gene *Ga05G2977* in hotspot 309 is bZIP transcription factor [36], and hotspot 309 was linked to a regulatory network of genes associated with cell wall biosynthesis and auxin response. It has been shown that *bZIP* genes play an important role in plant development and stress responses [32,37]. The homologous gene in Arabidopsis of *Ga05G2977* was ATbZIP18. It has been shown to regulate stem and root length by interacting with GA, and secondary cell wall thickening upon stimulation [38,39]. We demonstrate that the candidate gene *Ga05G2977* acts as an eQTL hotspot to regulate the expression of 325 genes that are involved in a regulatory network. Among those genes, 14 are associated with salt stress, and 29 are related to cell wall biosynthesis. In this group are three NAC transcription factors, NAT1 (*Ga01G1785*), NAC1 (*Ga11G2975*), and NAC36 (*Ga11G2976*). NST1 is regulated by ABA-mediated core signaling for abiotic stress and it orchestrates the transcriptional activation of secondary cell wall biosynthesis genes [33,40]. NAC36 is also induced by abiotic stresses, such as osmotic stress and salt stress, and mutation of this gene results in a dwarf phenotype in *Arabidopsis thaliana* [41]. Phospholipase D alpha 1 (PLDα1) affects seed germination and seedling development by ABA signaling and regulates callose deposition in response to salt [42,43,44]. The lignin biosynthesis genes *4CL1* and *PAL2* play critical roles under high-salt stress in *Arabidopsis thaliana* [38]. We hypothesize that the bZIP transcription factor protein *Ga05G2977* may play a vital role in cotton growth and salt tolerance through the regulation cell wall biosynthesis and salt stress.

Duplicated genes are a major source of new genes and biological functions [26]. In accordance with the present results, the majority of duplicated genes evolved under positive selection [27]. In this study, less than 10% of gene pairs were found to be conserved as eQTLs. According to the principle of repetitive gene sharing, the duplicated gene rapidly evolves to low expression to balance the dose effect of the gene [45]. *G. arboreum* is an ancient cotton species that has already completed short-term evolution, which may be the main reason for the low conservation of repetitive genes [46]. Therefore, we speculate that regulation of transcription is an important driver for changes in the function of duplicated genes [39]. However, the relationship between gene duplication and the evolution of transcriptional regulatory networks is largely unexplored. The tandemly duplicated genes in this study were found to be enriched for resistance-related functions, matching earlier studies for other species [47,48]. The percentage of the tandem genes regulated by eQTL was about 20%, but only 8% of tandemly duplicated gene pairs have identical eQTL. Previous studies have found that small-scale duplication events such as tandem duplications can disrupt the dose balance and tend to cause loss as opposed to preservation [45]. The results of this study showed that tandem genes might affect recent rapid evolution and spread of resistance to biological stress for cotton production [49].

We performed association analysis on the seedling stage transcriptome of *G. arboreum* populations and obtained the genes and larger genetic networks that determine cotton growth and salt tolerance. These insights into the genetic regulation of cotton growth and salt stress response can help provide a basis for a better understanding of cotton growth and stress tolerance.

## 4. Materials and Methods

### 4.1. Plant Materials

In a previous study, we constructed a genomic variation map by sequencing a natural population *of G. arboreum* accessions [25] To study the regulatory mechanisms of growth and salt resistance of cotton seedling, a total of 214 accessions were grown in a controlled greenhouse environment (in cycles of 16 h of light and 8 h of dark) during the spring of 2018 in Anyang (N 36.02°, E 114.50°). Each accession was grown in 8 plastic pots (3 seeds per pot) with vermiculite. We harvested the whole seedling at the two-leaf stage with one sample mix with five seedlings per sample for each accession. The samples were immediately frozen in liquid nitrogen after harvesting.

### 4.2. RNA Extraction and Sequencing

Total RNA was extracted using a Spectrum Plant Total RNA Kit (Sigma, STRN250). RNA quantities were checked by Qubit^®^ RNA Assay Kit in Qubit^®^ 2.0 Flurometer (Life Technologies, Carlsbad, CA, USA). RNA integrity was assessed using the RNA Nano 6000 Assay Kit of the Agilent Bioanalyzer 2100 system (Agilent Technologies, CA, USA). These RNA samples were then used to construct sequencing libraries using NEBNext^®^ UltraTM RNA Library Prep Kit for Illumina ^®^ (Carlsbad, CA, USA). cDNA fragments of 200–250 bp in length were selected with AMPure XP system (Beckman Coulter, Brea, CA, USA). Paired-end RNA sequencing of 150-bp reads was carried out on an Illumina HiSeq X Ten platform (paired-end 150 bp).

### 4.3. Differential Expression and Enrichment Analysis

Clean data from mRNA-seq were obtained by removing reads with adapter, reads with poly-N, and low quality reads of row data. The clean data were mapped to the reference genome sequence of *G. arboreum* [25] using the HISAT2 software with default settings [50]. The BAM files were used to calculate the FPKM (fragments per kilobase of exon model per million mapped fragments) of the different genes using the STRINGTIE software with default settings [51]. We counted the number of reads with the feature counts. We randomly selected twenty associations from each geographic and ecological group as experimental replicates. Differentially expressed genes (DEGs) were identified using the R package DESeq2 (V1.24.0) and the filtered results were adjusted with *p*-value of < 0.05 [52]. We repeated the two steps of selecting associations and DEGs analysis 30 times. The genes which were performed more than fifty times of the DEGs were regarded as the DEGs between any two of the three groups (i.e., SC versus YZR, SC versus YER, and YZR versus YER). The DEGs were submitted to the database for annotation, and richR (https://github.com/guokai8/richR accessed on 3 July 2021) was used to perform a Gene Ontology (GO) enrichment analysis and a Kyoto Encyclopedia of Genes and Genomes (KEGG) pathway enrichment analysis. A *p*-value < 0.05 was taken as indicating a statistically significant difference in both GO and KEGG analyses. 

### 4.4. Identification of eQTL

To identify eGWASs that are responsible for seedling development, we selected the genes whose expression was not detected only in less than 20% of the accessions. This allowed us to retain 28,382 of the 40,960 genes in the reference genome as expression traits for GWAS analysis. The unique mapping reads were parsed to identify single nucleotide polymorphisms (SNP) with SAMTOOLS and GATK (v.3.1.1) [53,54]. The 1,425,003 high-quality SNPs (MAF > 0.05, missing rate < 20%) were used to perform GWAS analysis. The population structure was calculated using the structure program and the kinship was derived from all SNPs. The 28,382 genes of expression level (FPKM) were regarded as the traits for GWAS analysis. We performed GWAS analysis for each gene using the EMMAX software [55]. The cutoff for filtering significant associations was 1.0 × 10^−5^ (1/n, where n is the effective number of independent SNPs).

The eQTL was identified under three conditions. The significant SNPs for each trait (gene) were grouped into clusters with a maximum distance of 100 kb between two consecutive SNPs. The putative eQTLs that were identified in a LD region (0.2) within 1 Mb were further filtered as false-positive associations. Finally, the clusters containing more than three significant SNPs were retained as putative eQTLs. Each eQTL was represented by the most significant SNP (lead SNP). In addition, to identify eQTL hotspots, we ran the HOT_SCAN program for all leader SNPs of eQTLs in each chromosome (−m 500 −s 0.05) [56].

### 4.5. GWAS on Seedling Quality-Related Traits

In this study, five salt tolerance related traits for cotton seedling under 0 and 150 mM NaCl treatments were analyzed, including seed germination rates (GR), fresh weight (FW), stem length (SL), water content (WC), and electric conductivity (EC) [57]. The relative values of the traits was calculated as RGR = GR_150_/GR_control_, i.e., the same as that for other traits, RFW, RSL, RWC, and REC. We performed a GWAS analysis using genomic variation data (1,425,003 SNPs) as those 214 accessions for eQTL analysis. The population structure was calculated using the structure program and the kinship was derived from all SNPs. We performed GWAS analysis for each gene using the EMMAX software [55]. The cutoff for determining significant associations was *p*-value < 1 × 10^−5^. We integrated the SNP by the eQTL loci as the QTL. The significant SNPs were grouped in the eQTL locus by its chromosome site. One QTL contains more than three significant SNP. 

### 4.6. TWAS

Transcriptome-wide association studies (TWAS) provided an approach for identifying significant cis-genetic correlation between expression and phenotypic traits [12]. It could be regarded as a test for non-zero local genetic correlation between expression and trait. We used a reference population with available gene expression and genetic variation data to impute the cis-genetic component of expression into another set of phenotype individuals where genetic variation has been identified. The reference was the genome-wide GWAS SNPs in those 214 cotton accessions for eQTL analysis. We performed a FUSION/TWAS (http://gusevlab.org/projects/fusion/ accessed on 3 July 2021) analysis on each chromosome containing significant GWAS associations for the traits of interest. The TWAS *p*-values were corrected for multiple testing using the Bonferroni correction method in R (v.3.6.3, Auckland, New Zealand).

### 4.7. Luciferase Assay in Nicotiana Benthamiana

To study the important role of the cis-eQTLs with the eGenes, we used the luciferase (luc) activity assay. The 939 bp Chr01G0472 promoter sequence was cloned into the plant binary vector pLL00R with a luciferase reporter gene (Table S12) [58]. After sequencing, the verified plasmids were transformed into GV3101. The *Agrobacterium harboring* reporter constructs were infiltrated into *N. benthamiana* leaves and 48 h later the luciferase signals were captured and analyzed using a Tanon 5200 Multi chemiluminescent imaging system (Tanon, Shanghai, China).

## Figures and Tables

**Table 1:** Identification of significant gene-trait associations in Gossypium arboreum using transcriptome-wide association study (TWAS).

Traits	ID	Homologue	BEST.GWAS.ID	EQTL.ID	TWAS.P	Annotation
FW_150_	*Ga05G0351*	AT2G31670	Chr05:2977850	Chr05:3010653	0.000181	stress responsive alpha-beta barrel domain protein, putative isoform 1
FW_150_	*Ga05G4258*	AT5G56040	Chr05:96415438	Chr05:96401623	0.000138	hypothetical protein F383_07100
FW_150_	*Ga05G4259*	AT3G13226	Chr05:96415438	Chr05:96631396	0.000724	regulatory recX
REC	*Ga10G0140*	AT5G17680	Chr10:1599353	Chr10:1462373	0.000663	leucine-rich repeat-containing protein
REC	*Ga10G0143*	AT4G12010	Chr10:1599353	Chr10:1455638	0.000501	hypothetical protein F383_19882
REC	*Ga05G1306*	AT3G15850	Chr05:11599695	Chr05:11595417	0.000207	fatty acid desaturase 5 isoform 1
SL_150_	*Ga03G0329*	AT3G09270	Chr03:3620246	Chr03:3621910	0.000325	glutathione transferase GST 23
SL_150_	*Ga03G0333*	AT3G24010	Chr03:3620246	Chr03:3656447	0.000193	RING/FYVE/PHD zinc finger superfamily protein isoform 1
SL_150_	*Ga03G0409*	AT4G14550	Chr03:4584192	Chr03:4572884	0.000606	auxin-responsive IAA14 -like protein
SL_150_	*Ga03G2393*	AT1G49980	Chr03:132884534	Chr03:132516809	0.000456	DNA/RNA polymerases superfamily protein isoform 1
WC_contral_	*Ga02G0459*	AT2G21860	Chr02:7132175	Chr02:7362933	0.00011	violaxanthin de-epoxidase, chloroplastic
WC_contral_	*Ga03G2524*	AT1G16700	Chr03:134024319	Chr03:133797580	4.06 × 10^−5^	hypothetical protein F383_06306
WC_contral_	*Ga07G2520*	AT5G14950	Chr07:95051631	Chr07:95051631	0.000128	golgi alpha-mannosidase II isoform 1
WC_150_	*Ga10G0152*	AT5G17680	Chr10:2324557	Chr10:2343608	4.13 × 10^−5^	hypothetical protein F383_19882
WC_150_	*Ga10G0163*	AT2G39940	Chr10:2324557	Chr10:2346135	0.000635	RNI-like superfamily protein
WC_150_	*Ga11G2207*	AT1G66120	Chr11:100108795	Chr11:100502650	0.000281	AMP-dependent synthetase and ligase family protein
WC_150_	*Ga11G3524*	AT5G65020	Chr11:119890411	Chr11:119605579	0.000788	annexin D2 -like protein
WC_150_	*Ga11G3887*	AT5G41770	Chr11:122024007	Chr11:122410413	0.000836	crooked neck-like protein 1
WC_150_	*Ga08G0064*	AT1G07410	Chr08:397149	Chr08:397149	6.83 × 10^−5^	RecName: Full = Ras-related protein Rab11C

## Data Availability

All raw genomic resequencing data for the 214 accessions have been deposited at in the NCBI BioProject database under accession number PRJNA349094. All of the raw sequencing data for mRNA-seq are accessible through NCBI under accession PRJNA704732.

## Supplementary Materials

The following supporting information can be downloaded at: https://www.mdpi.com/article/10.3390/ijms23147564/s1.
