# Supplementary material for: Integration of eQTL Analysis and GWAS Highlights Regulation Networks in Cotton under Stress Condition
Source: Int J Mol Sci. 2022 Jul 8;23(14):7564. doi: 10.3390/ijms23147564 (PMC9324452; doi:10.3390/ijms23147564)
Supplement: Supplementary file 1 [file ijms-23-07564-s001.zip › ijms-1786618-supplementary.pdf]

## Supplementary Figures

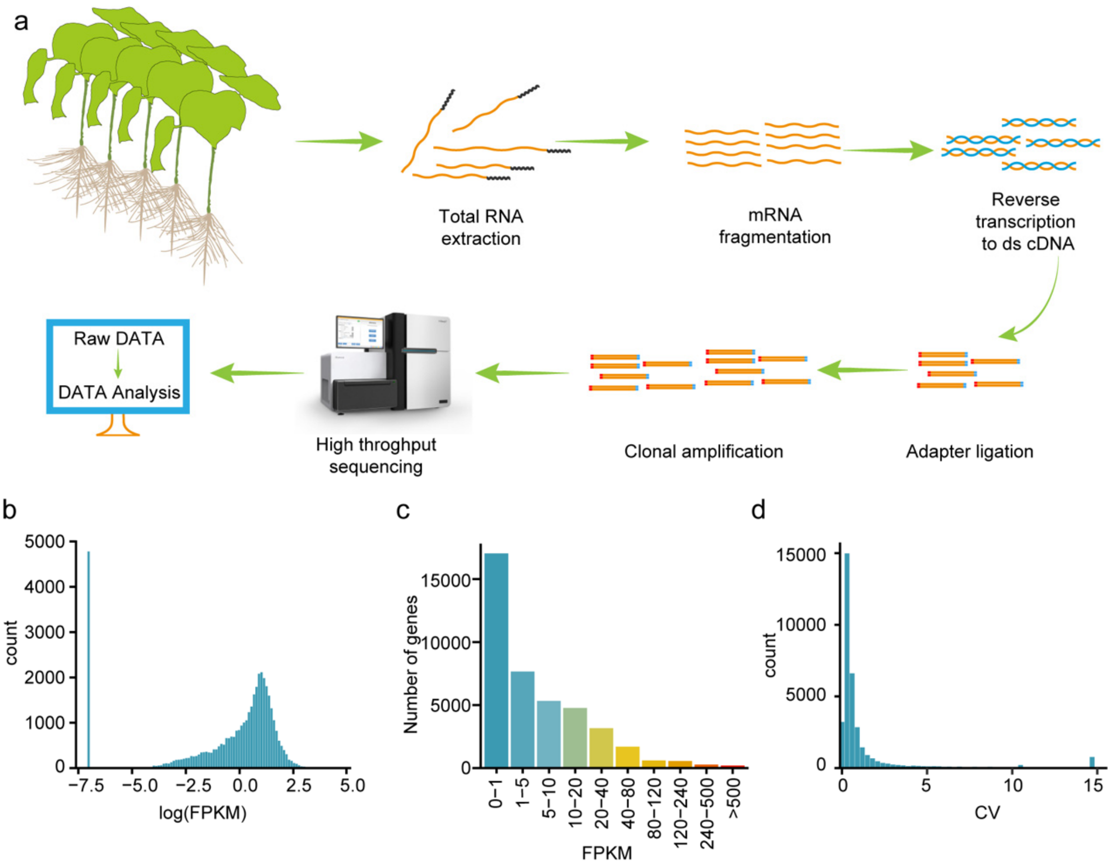


**Figure S1.** Overall statistics of RNA-Seq. (a) Analysis process for RNA-Seq (b) The histogram of log10 (FPKM). (c) FPKM statistic of genes. (d) The histogram of coefficient of variation (CV) for FPKM.

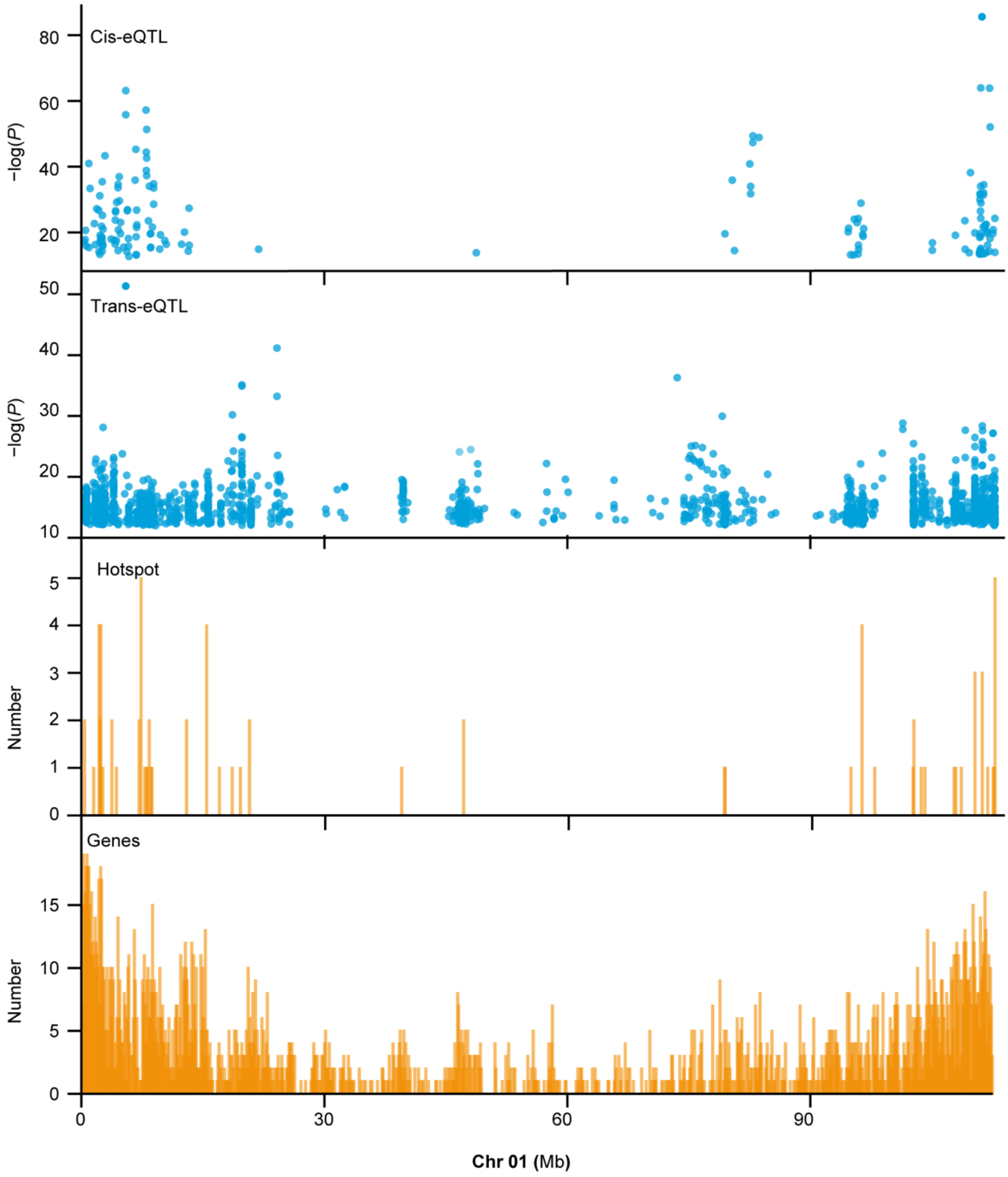


**Figure S2.** Integration of eQTLs and hotspots for chromosome 01. (a) Cis-eQTL ($p$-value < 1e-5) association plot. (b) Trans-eQTL ($p$-value < 1e-5) association plot. (c) Histogram of hotspots . (d) Density of gene in a 1 Mb window.

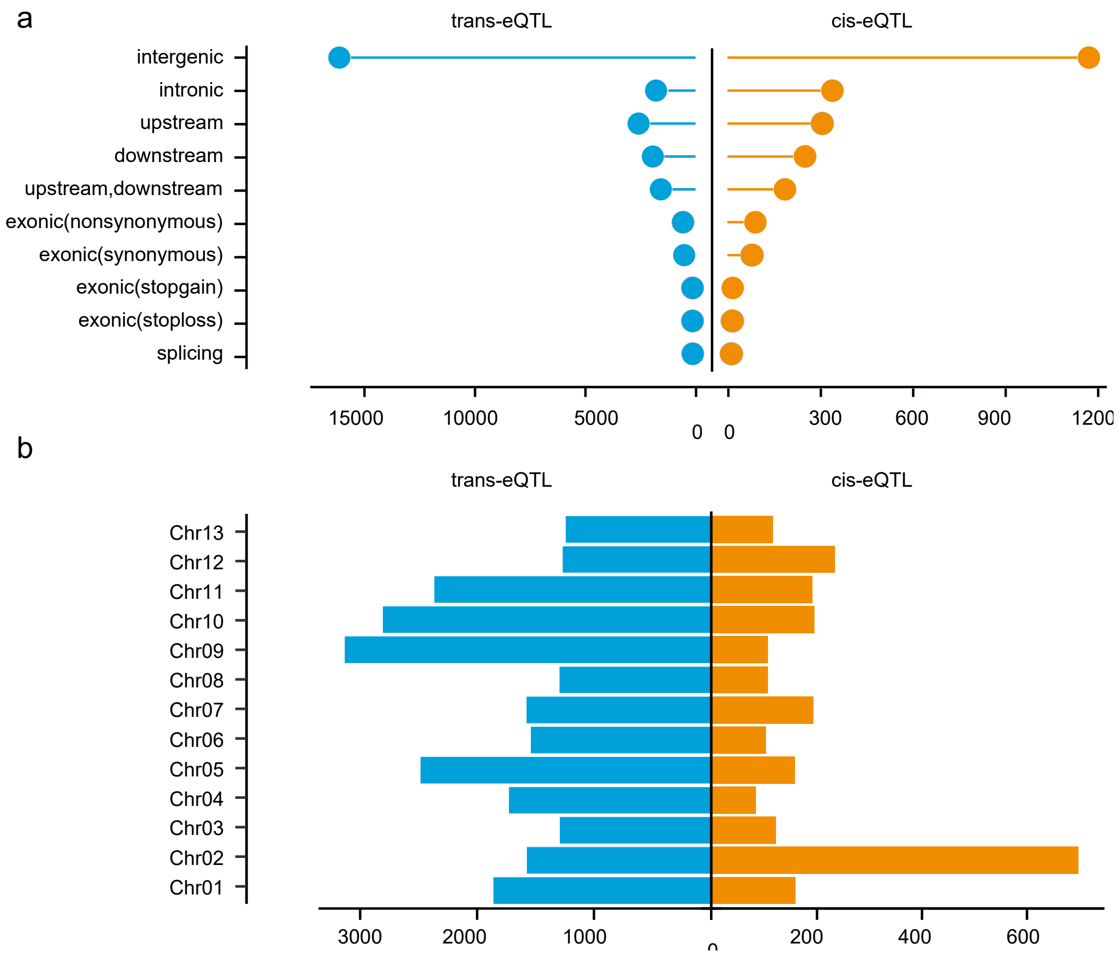


**Figure S3.** Number of cis-eQTLs and trans-eQTLs in different functional categories. (a) Number of cis-eQTLs and trans-eQTLs across functional categories. (b) Number of cis-eQTLs and trans-eQTLs in each chromosome.

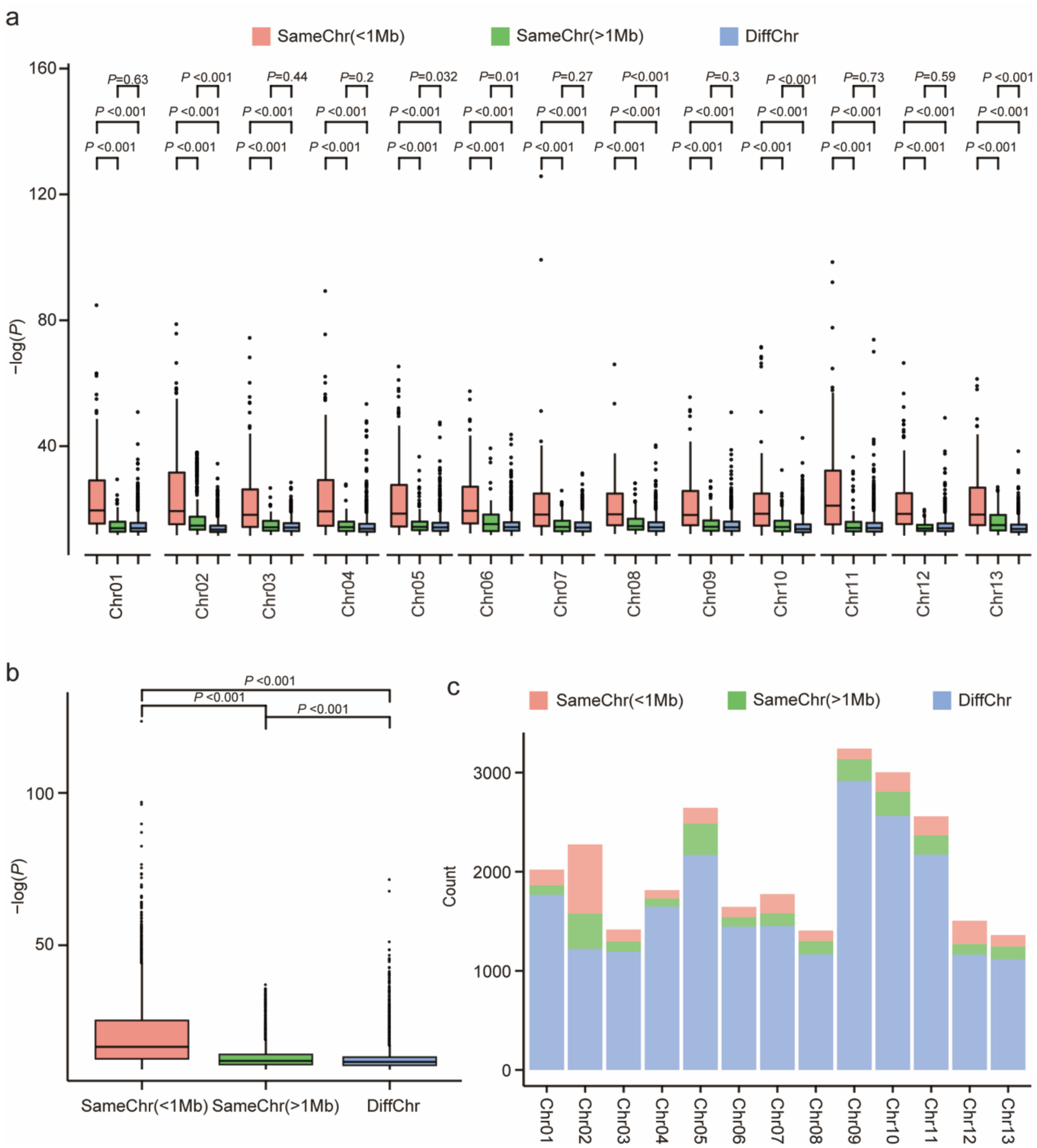


**Figure S4.** The variation in *p*-value for the distance between eQTL and eGene. (a) The comparison of significance (*p*-values) between intra-chromosomal eQTLs (SameChr) and inter-chromosomal eQTLs (DiffChr) for each chromosome. The intra-chromosomal eQTLs were divided into those with a distance of more than 1 Mb (SameChr (<1Mb)) and less than 1 Mb (SameChr (>1Mb)). (b) The comparison of significance (*p*-values) between SameChr (<1Mb), SameChr (>1Mb) and DiffChr. (c) The number of SameChr (<1Mb), SameChr (>1Mb) and DiffChr for each chromosome.

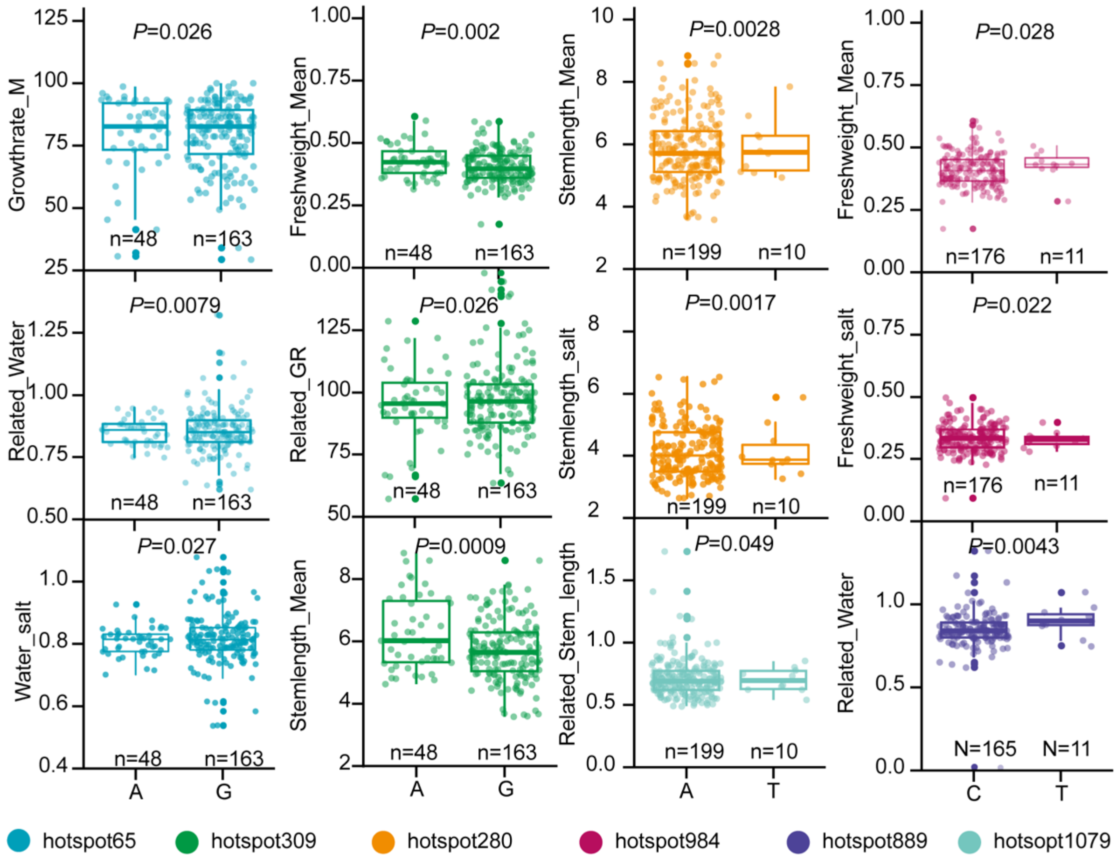


**Figure S5.** The significant divergence of the traits between different alleles of the lead SNP for 6 of the top 10 hotspots.

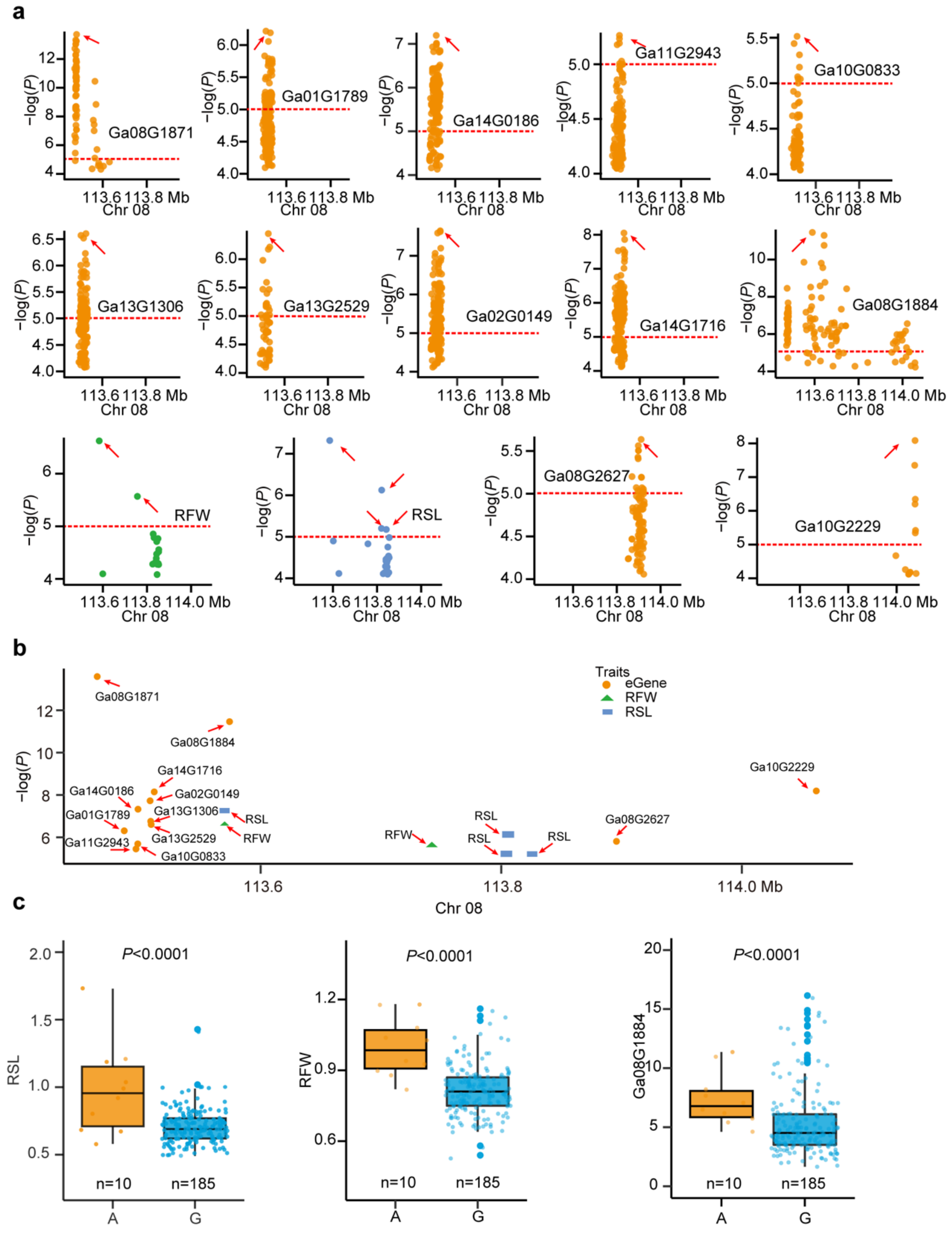


**Figure S6.** Integration of QTL and eQTL on Chr08:113453649-114064078. (a) Manhattan plot of GWAS and eGWAS signal on Chr08:113453649-114064078. (b) Manhattan plot of QTL and eQTL signal on Chr08:113453649-114064078. (c) The divergence of traits and expression of *Ga08G1884* between different alleles of the lead SNP.

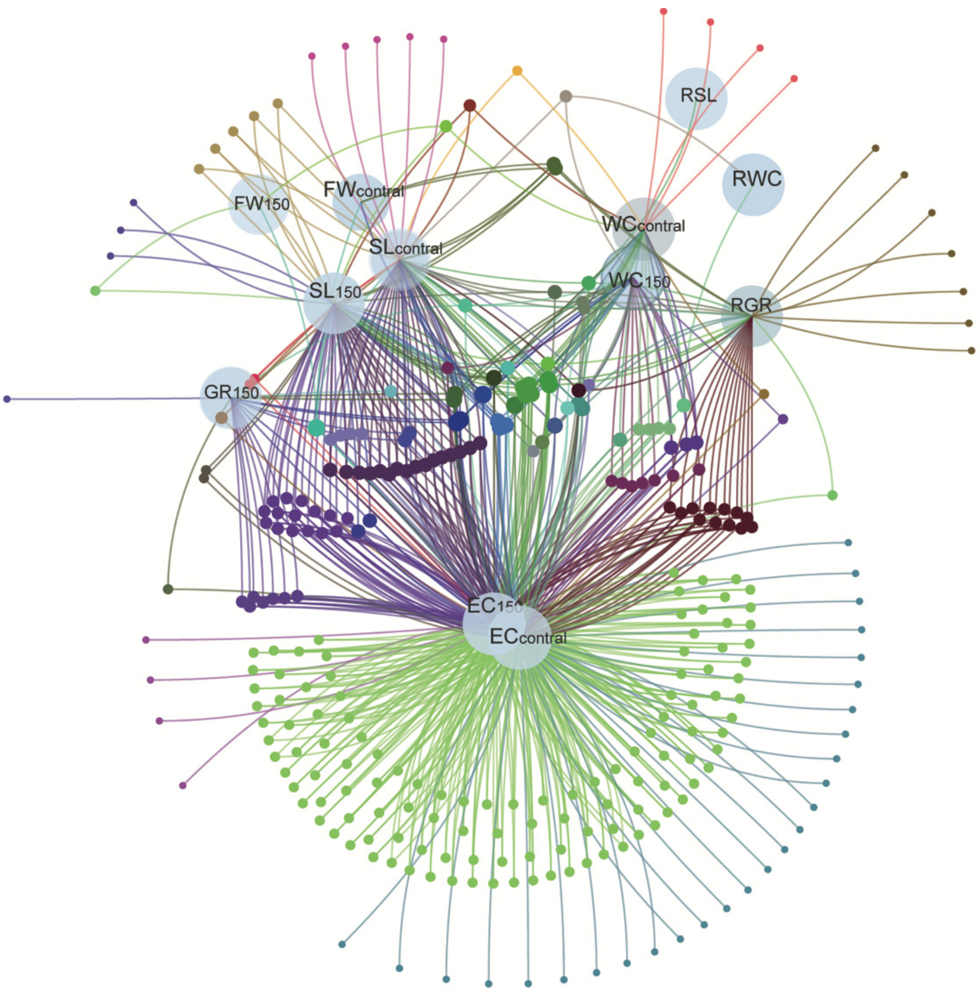


**Figure S7.** The network of traits and hotspot 309-related genes. The colorful points were hotspot 309-related genes. Grey points represented traits. Lines represent a significant correlation between the points.

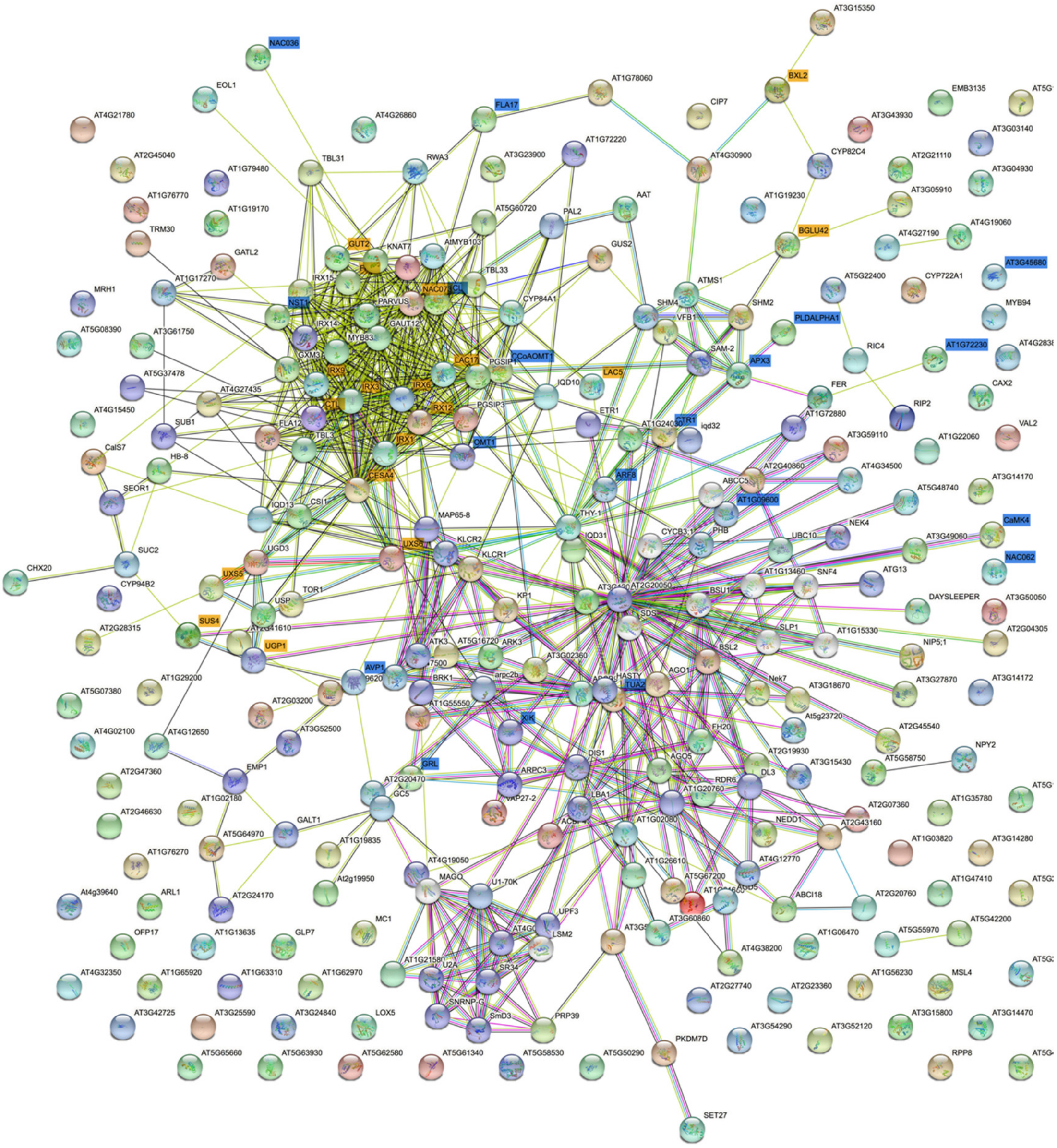

**Figure S8.** The network of *Arabidopsis thaliana* homologous genes for eGene of hotspot 309 by STRIGN. The genes highlighted in orange were homologous genes of cotton related with salt stress cotton. The genes highlighted in blue were homologous genes of cotton related with cell wall biosynthesis.

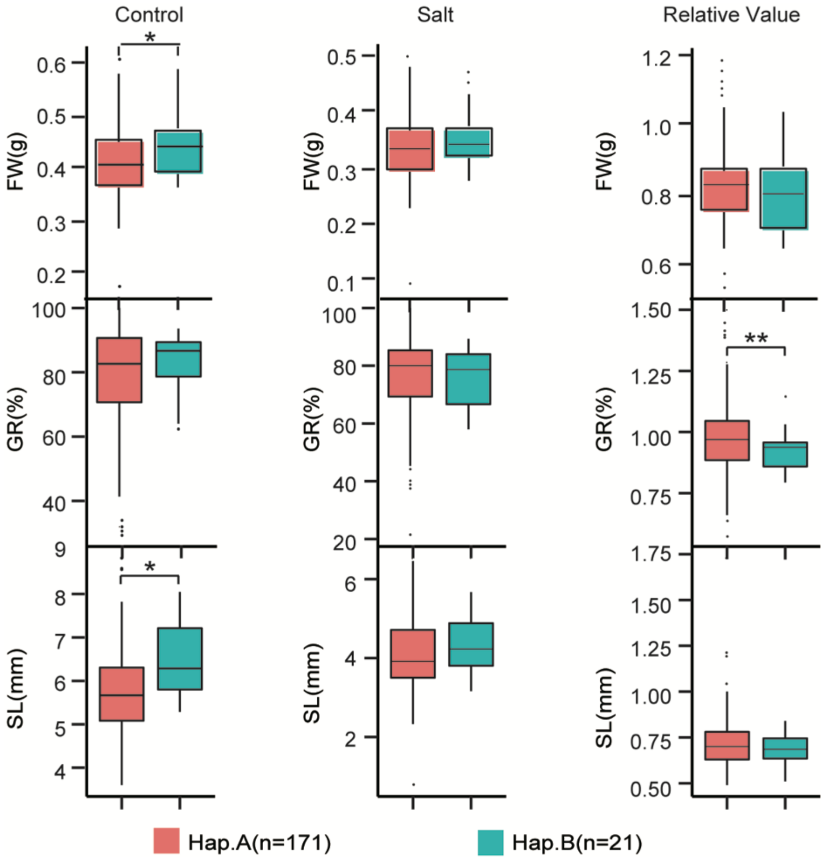


**Figure S9.** The traits of GRcontrol and GR150 was no different between Hap. B and Hap. A.

## Supplementary Tables

**Table S1.** Summary of RNA-Seq data in this study.

| sample | reads number | mapping |
|---|---|---|
| GA0001 | 36816366 | 91.05% |
| GA0002 | 27624118 | 92.08% |
| GA0003 | 29161426 | 93.93% |
| GA0005 | 30597874 | 94.77% |
| GA0006 | 34770050 | 95.19% |
| GA0007 | 29383329 | 94.86% |
| GA0008 | 27985385 | 95.25% |
| GA0009 | 37809408 | 92.93% |
| GA0010 | 28749746 | 95.07% |
| GA0011 | 29352912 | 93.55% |
| GA0012 | 28582015 | 92.07% |
| GA0013 | 27530729 | 94.90% |
| GA0014 | 27211068 | 94.38% |
| GA0015 | 28673820 | 93.98% |
| GA0016 | 28012329 | 95.22% |
| GA0017 | 29642195 | 94.86% |
| GA0018 | 29371273 | 94.37% |
| GA0019 | 30426101 | 95.39% |
| GA0020 | 27302226 | 95.41% |
| GA0021 | 27143086 | 94.42% |
| GA0022 | 27442714 | 94.39% |
| GA0023 | 27979518 | 92.62% |
| GA0096 | 34998821 | 93.55% |
| GA0097 | 37627028 | 90.70% |
| GA0098 | 37633402 | 89.98% |
| GA0099 | 38487522 | 89.47% |
| GA0100 | 30729013 | 91.00% |

| | | |
|---|---|---|
| GA0101 | 34703953 | 92.78% |
| GA0102 | 46015829 | 91.71% |
| GA0103 | 40047870 | 89.96% |
| GA0104 | 37047935 | 91.47% |
| GA0105 | 35231664 | 93.29% |
| GA0004 | 28110114 | 94.98% |
| GA0106 | 30940677 | 86.38% |
| GA0107 | 31837256 | 91.51% |
| GA0108 | 36707699 | 93.59% |
| GA0109 | 38002298 | 86.80% |
| GA0110 | 38098934 | 90.72% |
| GA0111 | 33691433 | 93.35% |
| GA0112 | 34862843 | 89.70% |
| GA0113 | 43259652 | 92.89% |
| GA0114 | 27569607 | 91.82% |
| GA0115 | 34947880 | 90.83% |
| GA0034 | 31362573 | 92.90% |
| GA0035 | 27978457 | 91.76% |
| GA0036 | 30324468 | 88.74% |
| GA0037 | 37026651 | 89.50% |
| GA0038 | 36707176 | 92.70% |
| GA0039 | 38244394 | 92.81% |
| GA0040 | 35855882 | 92.45% |
| GA0041 | 35642258 | 93.14% |
| GA0042 | 39751303 | 92.50% |
| GA0043 | 33589579 | 91.34% |
| GA0044 | 38710386 | 91.15% |
| GA0045 | 38056237 | 92.07% |
| GA0046 | 41714369 | 92.94% |
| GA0047 | 40854869 | 91.74% |
| GA0048 | 39893060 | 91.17% |
| GA0049 | 37710093 | 93.30% |
| GA0050 | 36907744 | 93.18% |
| GA0051 | 37635799 | 87.46% |
| GA0052 | 37345663 | 92.02% |
| GA0053 | 39325994 | 90.27% |
| GA0054 | 46201167 | 92.57% |
| GA0055 | 40075382 | 92.94% |
| GA0056 | 36907071 | 90.86% |
| GA0057 | 40925028 | 92.84% |
| GA0058 | 39719355 | 90.85% |
| GA0059 | 38112405 | 91.48% |
| GA0060 | 45385423 | 86.15% |
| GA0062 | 28502411 | 92.38% |
| GA0063 | 29538315 | 91.61% |
| GA0064 | 28116188 | 86.81% |
| GA0065 | 28708380 | 92.57% |
| GA0066 | 29939398 | 92.63% |
| GA0068 | 27883053 | 93.54% |
| GA0069 | 33254965 | 93.69% |
| GA0070 | 28449405 | 91.68% |
| GA0071 | 33031684 | 85.49% |
| GA0072 | 32443356 | 86.92% |
| GA0073 | 34511186 | 93.78% |
| GA0074 | 35953815 | 89.94% |
| GA0075 | 38427361 | 91.52% |
| GA0076 | 39241487 | 91.40% |
| GA0077 | 40169741 | 91.15% |
| GA0078 | 39564872 | 89.66% |
| GA0079 | 40530657 | 89.87% |
| GA0080 | 35888447 | 87.15% |
| GA0081 | 32856829 | 88.11% |

| | | |
|---|---|---|
| GA0082 | 35709213 | 88.77% |
| GA0083 | 33972715 | 89.99% |
| GA0084 | 36107206 | 89.53% |
| GA0085 | 33727984 | 90.87% |
| GA0086 | 32782221 | 89.18% |
| GA0087 | 31842695 | 87.26% |
| GA0088 | 32804267 | 86.70% |
| GA0089 | 37232428 | 91.22% |
| GA0090 | 35365881 | 93.35% |
| GA0091 | 35673949 | 92.58% |
| GA0093 | 32222235 | 91.73% |
| GA0094 | 36483851 | 87.11% |
| GA0095 | 32306255 | 93.23% |
| GA0116 | 34363921 | 85.82% |
| GA0117 | 34314547 | 93.93% |
| GA0118 | 26921467 | 92.13% |
| GA0119 | 28945523 | 86.08% |
| GA0120 | 32103578 | 86.22% |
| GA0121 | 40609699 | 92.74% |
| GA0122 | 34177991 | 93.34% |
| GA0123 | 31966417 | 89.65% |
| GA0124 | 32373243 | 93.84% |
| GA0125 | 27660321 | 91.44% |
| GA0126 | 30995817 | 91.45% |
| GA0127 | 38680605 | 89.21% |
| GA0128 | 30180943 | 92.12% |
| GA0129 | 28971001 | 88.36% |
| GA0130 | 36669541 | 92.57% |
| GA0131 | 34188453 | 88.03% |
| GA0132 | 28584780 | 92.61% |
| GA0133 | 27712691 | 88.75% |
| GA0134 | 36058275 | 93.17% |
| GA0135 | 30205236 | 92.83% |
| GA0136 | 33453647 | 91.71% |
| GA0137 | 35085736 | 91.68% |
| GA0138 | 27462946 | 86.11% |
| GA0139 | 31644257 | 92.40% |
| GA0140 | 35834877 | 93.09% |
| GA0141 | 31601199 | 92.72% |
| GA0142 | 33395176 | 86.51% |
| GA0143 | 33562054 | 89.50% |
| GA0144 | 34090980 | 87.26% |
| GA0145 | 34475799 | 87.90% |
| GA0146 | 38980372 | 93.88% |
| GA0147 | 34690064 | 91.80% |
| GA0148 | 34412396 | 91.07% |
| GA0149 | 31617212 | 92.34% |
| GA0150 | 33200184 | 86.64% |
| GA0151 | 33464967 | 89.17% |
| GA0152 | 34716070 | 92.69% |
| GA0153 | 36980106 | 91.48% |
| GA0154 | 29950302 | 90.76% |
| GA0155 | 30146170 | 91.84% |
| GA0156 | 43815201 | 91.81% |
| GA0157 | 35657387 | 93.30% |
| GA0158 | 36979166 | 89.88% |
| GA0159 | 37081754 | 89.85% |
| GA0160 | 35203085 | 89.99% |
| GA0161 | 34982943 | 91.45% |
| GA0162 | 35098388 | 86.35% |
| GA0163 | 33154216 | 87.21% |
| GA0164 | 37720850 | 92.42% |

| | | |
|---|---|---|
| GA0165 | 33656097 | 93.03% |
| GA0166 | 43234366 | 93.54% |
| GA0167 | 35445130 | 91.38% |
| GA0168 | 34896901 | 92.18% |
| GA0169 | 31701837 | 86.63% |
| GA0170 | 35454473 | 92.46% |
| GA0171 | 36548774 | 90.90% |
| GA0172 | 35964221 | 86.78% |
| GA0173 | 32958405 | 91.28% |
| GA0174 | 36093498 | 93.77% |
| GA0175 | 32346400 | 94.13% |
| GA0177 | 34080403 | 94.59% |
| GA0178 | 37477894 | 93.75% |
| GA0179 | 38979785 | 92.62% |
| GA0180 | 39782899 | 90.93% |
| GA0181 | 36243827 | 94.46% |
| GA0182 | 34728905 | 92.49% |
| GA0183 | 33852190 | 93.86% |
| GA0184 | 32243716 | 93.86% |
| GA0185 | 40508985 | 93.43% |
| GA0187 | 43693474 | 93.04% |
| GA0188 | 33900121 | 93.48% |
| GA0189 | 37520624 | 93.38% |
| GA0190 | 37187243 | 92.99% |
| GA0191 | 40769105 | 93.64% |
| GA0192 | 37226612 | 93.83% |
| GA0193 | 39538512 | 94.41% |
| GA0194 | 38599061 | 94.24% |
| GA0195 | 38277866 | 94.05% |
| GA0206 | 38692208 | 93.48% |
| GA0207 | 36030138 | 93.14% |
| GA0208 | 42088024 | 89.98% |
| GA0209 | 28206635 | 94.24% |
| GA0210 | 33423557 | 93.59% |
| GA0211 | 39317230 | 94.12% |
| GA0213 | 36937665 | 93.56% |
| GA0214 | 40253351 | 93.77% |
| GA0215 | 35022673 | 93.80% |
| GA0061 | 31599756 | 94.35% |
| GA0092 | 31698387 | 93.62% |
| GA0176 | 35695185 | 93.64% |
| GA0186 | 40509184 | 93.41% |
| GA0024 | 32975556 | 93.13% |
| GA0025 | 31019076 | 92.08% |
| GA0026 | 32219035 | 92.79% |
| GA0027 | 34323257 | 90.71% |
| GA0028 | 26788020 | 92.66% |
| GA0029 | 29025456 | 87.48% |
| GA0030 | 32184692 | 92.12% |
| GA0031 | 32047039 | 90.81% |
| GA0032 | 27844483 | 92.20% |
| GA0033 | 29550015 | 91.60% |
| GA0196 | 38004168 | 92.63% |
| GA0197 | 35150437 | 93.08% |
| GA0198 | 39082544 | 93.84% |
| GA0199 | 33778640 | 92.99% |
| GA0200 | 32984044 | 93.85% |
| GA0201 | 34147075 | 94.65% |
| GA0202 | 33467173 | 94.20% |
| GA0203 | 34964569 | 93.22% |
| GA0204 | 35217953 | 93.86% |
| GA0205 | 42775437 | 92.80% |

**Table S2.** Gene expression statistics.

| FPKM | Number of genes | Percentage |
|---|---|---|
| 0-1 | 16935 | 41.35% |
| 1-5 | 7609 | 18.58% |
| 5-10 | 5293 | 12.92% |
| 10-20 | 4738 | 11.57% |
| 20-40 | 3135 | 7.65% |
| 40-80 | 1678 | 4.10% |
| 80-120 | 581 | 1.42% |
| 120-240 | 543 | 1.33% |
| 240-500 | 262 | 0.64% |
| >500 | 186 | 0.45% |

**Table S3.** Statistics of significant SNP associated with treatments.

| **Control treatment** | | **Salt treatment** | | Relative treatment | |
|---|---|---|---|---|---|
| **Treatment** | **Number** | **Treatment** | Number | Treatment | number |
| $GR_{control}$ | 12 | $GR_{150}$ | 4 | RGR | 0 |
| $FW_{control}$ | 22 | $FW_{150}$ | 989 | RFW | 20 |
| $SL_{control}$ | 39 | $SL_{150}$ | 57 | RSL | 40 |
| WCcontrol | 634 | $WC_{150}$ | 387 | RWC | 25 |
| $EC_{control}$ | 1874 | $EC_{150}$ | 49 | REC | 8 |

**Table S4.** QTL(Chr08:113453649-114064078)-related gene expression and traits.

| QTL Type | Traits | CHR | Site | Pvalue | Type | GeneID Annotation | reference |
|---|---|---|---|---|---|---|---|
| | Ga01G1789 | Chr08 | 113484533 | 7.24E-07 | Trans-eQTLs | Disease resistance-responsive family protein [Theobroma cacao] | Faisal et.al 2020 |
| | Ga02G0149 | Chr08 | 113506218 | 2.84E-08 | Trans-eQTLs | Pyridine nucleotide-disulfide oxidoreductase domain-containing 2 [Gossypium arboreum] | |
| | Ga04G1991 | Chr08 | 113483025 | 1.91E-06 | Trans-eQTLs | Glycerol-3-phosphate dehydrogenase [NAD(P)+] [Gossypium arboreum] | Katarzyna et.al 2012 |
| | Ga08G1871 | Chr08 | 113462536 | 3.57E-14 | Cis-eQTLs | Nitrate transporter 1.5 -like protein [Gossypium arboreum] | Hong et.al 2017 |
| eQTL | Ga08G1873 | Chr08 | 113484348 | 1.26E-06 | Cis-eQTLs | Phosphoribosyltransferase family protein [Theobroma cacao] | |
| | Ga08G1884 | Chr08 | 113571400 | 5.70E-12 | Cis-eQTLs | Actin-regulating kinase PRK1 [Gossypium arboreum] | Yong-hua et.al 2018 |
| | Ga08G2627 | Chr08 | 113892910 | 2.57E-06 | Trans-eQTLs | Leucoanthocyanidin dioxygenase, putative [Theobroma cacao] | Ilya et.al 2006;Muhammad et.al 2020 |
| | Ga10G0833 | Chr08 | 113495059 | 3.37E-06 | Trans-eQTLs | Asparticase nepenthesin-1 [Gossypium arboreum] | Behnaz et.al 2013; Anna et.al 2015 |
| | Ga10G2229 | Chr08 | 114059004 | 1.07E-08 | Trans-eQTLs | 2,3-bisphosphoglycerate-dependent phosphoglycerate mutase [Gossypium arboreum] | Ming et.al 2015 |

| | | | | | | | |
|---|---|---|---|---|---|---|---|
| | Ga11G2943 | Chr08 | 113493618 | 5.91E-06 | Trans-eQTLs | Transcription factor PIF3 -like protein [Gossypium arboreum] | Trijatmiko et.al 2004; Barah et.al 2013 |
| | Ga13G1306 | Chr08 | 113505818 | 2.93E-07 | Trans-eQTLs | hypothetical protein F383_37108 [Gossypium arboreum] | Xiao et.al 2013 |
| | Ga13G1920 | Chr08 | 113480420 | 1.34E-06 | Trans-eQTLs | Arginine/serine-rich 45 [Theobroma cacao] | Albaqami et.al 2019 |
| | Ga13G1949 | Chr08 | 113497997 | 2.71E-06 | Trans-eQTLs | Cytochrome P450 71D10, putative [Theobroma cacao] | Kim et.al 2019 |
| | Ga13G2529 | Chr08 | 113506224 | 4.15E-07 | Trans-eQTLs | hypothetical protein F383_01057 [Gossypium arboreum] | |
| | Ga14G0186 | Chr08 | 113495295 | 7.84E-08 | Trans-eQTLs | hypothetical protein F383_14723 [Gossypium arboreum] | |
| | Ga14G1716 | Chr08 | 113509512 | 1.15E-08 | Trans-eQTLs | hypothetical protein F383_08088 [Gossypium arboreum] | |
| QTL | Related_Stem_length | Chr08 | 113567118 | 5.98E-08 | | | |
| | Related_Stem_length | Chr08 | 113801247 | 7.77E-06 | | | |
| | Related_Stem_length | Chr08 | 113802555 | 9.34E-07 | | | |
| | Related_Stem_length | Chr08 | 113823174 | 8.30E-06 | | | |

**Table S5.** Correlation between the traits of the QTL (Chr08:113453649-114064078).

| Traits 1 | Traits 2 | Correlation(Pearson) | Pvalue |
|---|---|---|---|
| Ga02G0149 | Ga01G1789 | 0.24258691 | 0.00035274 |
| Ga13G1306 | Ga01G1789 | 0.17722032 | 0.00954827 |
| Ga13G1920 | Ga01G1789 | 0.43395495 | 3.42E-11 |
| Ga13G1949 | Ga01G1789 | 0.3989369 | 1.53E-09 |
| Ga13G2529 | Ga01G1789 | 0.40774376 | 6.14E-10 |
| Ga14G0186 | Ga01G1789 | 0.53342923 | 4.63E-17 |
| Ga14G1716 | Ga01G1789 | 0.29911842 | 8.92E-06 |
| Ga08G1873 | Ga02G0149 | 0.16903908 | 0.01349884 |
| Ga10G2229 | Ga02G0149 | 0.31985959 | 1.87E-06 |
| Ga13G1306 | Ga02G0149 | 0.27070606 | 6.27E-05 |
| Ga13G1920 | Ga02G0149 | 0.31200262 | 3.43E-06 |
| Ga13G2529 | Ga02G0149 | 0.21775474 | 0.00138487 |
| Ga14G0186 | Ga02G0149 | 0.34217145 | 3.06E-07 |
| Ga08G2627 | Ga04G1991 | -0.152191 | 0.02634836 |
| Ga11G2943 | Ga04G1991 | 0.40119076 | 1.22E-09 |
| Ga13G1306 | Ga04G1991 | 0.36606202 | 3.73E-08 |
| Ga13G1920 | Ga04G1991 | 0.39055504 | 3.58E-09 |
| Ga13G1949 | Ga04G1991 | 0.37626841 | 1.44E-08 |
| Ga13G2529 | Ga04G1991 | 0.29688009 | 1.05E-05 |
| Ga14G0186 | Ga04G1991 | 0.36163173 | 5.58E-08 |
| Ga14G1716 | Ga04G1991 | 0.25623831 | 0.00015621 |
| Ga08G1873 | Ga08G1871 | 0.19143813 | 0.00505539 |
| Ga10G2229 | Ga08G1871 | 0.30389987 | 6.29E-06 |
| Ga13G1306 | Ga08G1871 | 0.23552156 | 0.0005283 |

| | | | |
|---|---|---|---|
| Ga13G1920 | Ga08G1871 | 0.22466948 | 0.00096011 |
| Ga14G0186 | Ga08G1871 | 0.18254584 | 0.0075632 |
| Ga13G1920 | Ga08G1873 | 0.15979455 | 0.01962636 |
| Ga13G1949 | Ga08G1873 | 0.16728075 | 0.01451481 |
| Ga13G2529 | Ga08G1873 | 0.32345433 | 1.41E-06 |
| Ga14G1716 | Ga08G1873 | 0.27835607 | 3.78E-05 |
| Ga10G2229 | Ga08G1884 | 0.13501228 | 0.0490837 |
| Ga13G1306 | Ga08G1884 | 0.40098941 | 1.24E-09 |
| Ga13G1920 | Ga08G1884 | 0.38894145 | 4.20E-09 |
| Ga13G1949 | Ga08G1884 | 0.37794282 | 1.23E-08 |
| Ga13G2529 | Ga08G1884 | 0.33931789 | 3.89E-07 |
| Ga14G1716 | Ga08G1884 | 0.35477938 | 1.03E-07 |
| Ga10G2229 | Ga08G2627 | -0.1356092 | 0.04808173 |
| Ga10G2229 | Ga10G0833 | 0.13635845 | 0.04684829 |
| Ga13G1306 | Ga10G0833 | 0.49994094 | 7.19E-15 |
| Ga13G1920 | Ga10G0833 | 0.7108621 | 4.33E-34 |
| Ga13G1949 | Ga10G0833 | 0.51827425 | 4.87E-16 |
| Ga13G2529 | Ga10G0833 | 0.50752419 | 2.41E-15 |
| Ga14G0186 | Ga10G0833 | 0.60652279 | 8.67E-23 |
| Ga14G1716 | Ga10G0833 | 0.45280973 | 3.65E-12 |
| Ga13G1920 | Ga10G2229 | 0.18620436 | 0.00642141 |
| Ga14G0186 | Ga10G2229 | 0.20749966 | 0.00233661 |
| Ga13G1306 | Ga11G2943 | 0.44305894 | 1.18E-11 |
| Ga13G1949 | Ga11G2943 | 0.56970834 | 1.00E-19 |
| Ga13G2529 | Ga11G2943 | 0.28323386 | 2.72E-05 |
| Ga14G0186 | Ga11G2943 | 0.2746939 | 4.83E-05 |
| Ga14G1716 | Ga11G2943 | 0.34817399 | 1.83E-07 |
| Ga13G1949 | Ga13G1306 | 0.32805238 | 9.79E-07 |
| Ga13G2529 | Ga13G1306 | 0.27822601 | 3.82E-05 |
| Ga14G0186 | Ga13G1306 | 0.35556018 | 9.61E-08 |
| Ga14G1716 | Ga13G1306 | 0.34884097 | 1.73E-07 |
| Ga11G2943 | Ga13G1920 | 0.32119804 | 1.69E-06 |
| Ga13G1949 | Ga13G1920 | 0.4130495 | 3.49E-10 |
| Ga13G2529 | Ga13G1920 | 0.41860018 | 1.91E-10 |
| Ga14G0186 | Ga13G1920 | 0.6091201 | 5.09E-23 |
| Ga14G1716 | Ga13G1920 | 0.29198557 | 1.48E-05 |
| Ga13G2529 | Ga13G1949 | 0.58596373 | 4.97E-21 |
| Ga14G0186 | Ga13G1949 | 0.4512425 | 4.42E-12 |
| Ga14G1716 | Ga13G1949 | 0.40957557 | 5.06E-10 |
| Related_FreshW | Ga13G1949 | -0.1534525 | 0.0261719 |
| Ga14G0186 | Ga13G2529 | 0.51102794 | 1.44E-15 |
| Ga14G1716 | Ga13G2529 | 0.45822352 | 1.87E-12 |
| Ga14G1716 | Ga14G0186 | 0.34294214 | 2.87E-07 |
| Related_FreshW | Related_Stem_length | 0.70119252 | 2.16E-32 |

**Table S6.** Gene ontology (GO) enrichment of hotspot 309.

| Annot | Term | Annotated | Significant | *p*-value | Padj | GeneID |
|---|---|---|---|---|---|---|
| GO:0009834 | plant-type secondary cell wall biogenesis | 8 | 5 | 8.40E-09 | 2.18E-07 | Ga03G2054,Ga07G2381,Ga11G2338,Ga11G3582,Ga13G2817 |
| GO:0006413 | translational initiation | 16 | 3 | 0.0007157 | 0.00930407 | Ga09G1034,Ga09G1869,Ga12G0044 |
| GO:0006464 | cellular protein modification process | 7 | 2 | 0.00263061 | 0.0227986 | Ga12G0229,Ga13G2148 |
| GO:0009698 | phenylpropanoid metabolic process | 1 | 1 | 0.0115626 | 0.05945243 | Ga10G2561 |
| GO:0010106 | cellular response to iron ion starvation | 1 | 1 | 0.0115626 | 0.05945243 | Ga08G1251 |

| | | | | | | |
|---|---|---|---|---|---|---|
| GO:0032259 | methylation | 28 | 2 | 0.04068614 | 0.06611498 | Ga08G1237,Ga12G0336 |

**Table S7.** KEGG enrichment analysis of hotspots309.

| Annot | Term | Annotated | Significant | *p*-value | Padj | GeneID |
|---|---|---|---|---|---|---|
| ko04144 | Endocytosis | 215 | 7 | 0.0003702 | 0.01193726 | Ga03G0161,Ga04G0145,Ga07G0340,Ga08G0374,Ga10G1624,Ga10G3060,Ga13G1791 |
| ko00940 | Phenylpropanoid biosynthesis | 232 | 7 | 0.00058551 | 0.01193726 | Ga04G0313,Ga08G1237,Ga10G0561,Ga10G2561,Ga11G0347,Ga12G0336,Ga11G2107 |
| ko00460 | Cyanoamino acid metabolism | 65 | 4 | 0.00072495 | 0.01193726 | Ga10G1527,Ga11G0347,Ga11G3514,Ga14G1502 |
| ko00670 | One carbon pool by folate | 31 | 3 | 0.00094838 | 0.01193726 | Ga03G2541,Ga10G1527,Ga14G1502 |
| ko00520 | Amino sugar and nucleotide sugar metabolism | 189 | 6 | 0.00114781 | 0.01193726 | Ga01G0796,Ga02G0822,Ga08G0443,Ga12G1218,Ga12G2624,Ga13G1828 |
| ko00500 | Starch and sucrose metabolism | 316 | 7 | 0.00348672 | 0.0302182 | Ga01G0796,Ga05G0384,Ga06G0858,Ga08G0443,Ga11G0347,Ga12G1218,Ga13G1828 |
| ko00053 | Ascorbate and aldarate metabolism | 70 | 3 | 0.00971711 | 0.07218426 | Ga02G0079,Ga02G0822,Ga12G1218 |
| ko00360 | Phenylalanine metabolism | 153 | 4 | 0.01565352 | 0.10174791 | Ga04G0313,Ga10G0561,Ga10G2561,Ga11G1604 |
| ko00480 | Glutathione metabolism | 121 | 3 | 0.04087949 | 0.23619259 | Ga02G0079,Ga08G0610,Ga11G3514 |

**Table S8.** KEGG ontology (KO) enrichment analysis of hotspot309.

| Annot | Term | Annotated | Significant | *p*-value | Padj | GeneID |
|---|---|---|---|---|---|---|
| K10999 | K10999 cellulose synthase A [EC:2.4.1.12] (A) | 18 | 5 | 3.40E-07 | 2.69E-05 | Ga05G0095,Ga07G0463,Ga07G2381,Ga08G0544,Ga10G2710 |
| K11593 | K11593 eukaryotic translation initiation factor 2C (A) | 15 | 4 | 6.62E-06 | 0.00026163 | Ga09G1032,Ga09G1034,Ga09G1869,Ga12G0044 |
| K10406 | K10406 kinesin family member C2/C3 (A) | 21 | 4 | 2.79E-05 | 0.00073485 | Ga01G1003,Ga07G1406,Ga08G1402,Ga11G0822 |
| K08857 | K08857 NIMA (never in mitosis gene a)-related kinase [EC:2.7.11.1] (A) | 12 | 3 | 0.00012942 | 0.00247674 | Ga02G1456,Ga08G0755,Ga09G1422 |
| K15285 | K15285 solute carrier family 35, member E3 (A) | 13 | 3 | 0.00016719 | 0.00247674 | Ga01G2415,Ga05G1100,Ga08G2334 |
| K02184 | K02184 formin 2 (A) | 3 | 2 | 0.00021946 | 0.00247674 | Ga11G3744,Ga12G1033 |
| K16547 | K16547 protein NEDD1 (A) | 3 | 2 | 0.00021946 | 0.00247674 | Ga10G0239,Ga10G0241 |
| K17086 | K17086 transmembrane 9 superfamily member 2/4 (A) | 18 | 3 | 0.00046227 | 0.00456492 | Ga05G0576,Ga05G0636,Ga14G2234 |

| | | | | | | |
|---|---|---|---|---|---|---|
| K12471 | K12471 epsin (A) | 5 | 2 | 0.0007233 | 0.006349 | Ga03G0161,Ga10G1624 |
| K11699 | K11699 RNA-dependent RNA polymerase [EC:2.7.7.48] (A) | 6 | 2 | 0.00107885 | 0.00852289 | Ga05G0583,Ga07G0092 |
| K18442 | K18442 brefeldin A-inhibited guanine nucleotide-exchange protein (A) | 8 | 2 | 0.00199126 | 0.01430083 | Ga04G0145,Ga08G0374 |
| K00695 | K00695 sucrose synthase [EC:2.4.1.13] (A) | 10 | 2 | 0.00316437 | 0.02083213 | Ga05G0384,Ga06G0858 |
| K05750 | K05750 NCK-associated protein 1 (A) | 1 | 1 | 0.00861539 | 0.04272842 | Ga14G2625 |
| K07999 | K07999 matrix metalloproteinase-20 (enamelysin) [EC:3.4.24.-] (A) | 1 | 1 | 0.00861539 | 0.04272842 | Ga11G3250 |
| K11093 | K11093 U1 small nuclear ribonucleoprotein 70kDa (A) | 1 | 1 | 0.00861539 | 0.04272842 | Ga10G2030 |
| K00600 | K00600 glycine hydroxymethyltransferase [EC:2.1.2.1] (A) | 17 | 2 | 0.00919472 | 0.04272842 | Ga10G1527,Ga14G1502 |
| K11000 | K11000 callose synthase [EC:2.4.1.-] (A) | 17 | 2 | 0.00919472 | 0.04272842 | Ga04G1728,Ga14G1699 |
| K09422 | K09422 myb proto-oncogene protein, plant (A) | 216 | 6 | 0.01092831 | 0.0479631 | Ga01G2601,Ga08G1352,Ga11G4112,Ga12G0584,Ga12G1064,Ga13G2645 |
| K13066 | K13066 caffeic acid 3-O-methyltransferase [EC:2.1.1.68] (A) | 24 | 2 | 0.01794376 | 0.04888128 | Ga08G1237,Ga12G0336 |
| K13648 | K13648 alpha-1,4-galacturonosyltransferase [EC:2.4.1.43] (A) | 25 | 2 | 0.01939565 | 0.05107521 | Ga01G0796,Ga13G1828 |
| K09338 | K09338 homeobox-leucine zipper protein (A) | 68 | 3 | 0.02080695 | 0.05302417 | Ga05G1392,Ga07G0275,Ga10G3057 |

**Table S9.** Cell wall biosynthesis-related genes and salt stress response of hotspot309.

| GeneID | Type | Gene Name | nr annotation | Model name | Short description |
|---|---|---|---|---|---|
| Ga05G1026 | cellwall | UXS6 | UDP-glucuronic acid decarboxylase 1 [Gossypium arboreum] | AT2G28760.3 | UDP-XYL synthase 6 |
| Ga01G2357 | cellwall | UXS5 | UDP-glucuronic acid decarboxylase 1 [Gossypium arboreum] | AT3G46440.2 | UDP-XYL synthase 5 |
| Ga08G0443 | cellwall | UGPA | UTP--glucose-1-phosphate uridylyltransferase [Gossypium arboreum] | AT3G03250.1 | UDP-GLUCOSE PYROPHOSPHORYLASE 1 |
| Ga05G0384 | cellwall | SS | sucrose synthase 3 [Gossypium arboreum] | AT3G43190.1 | sucrose synthase 4 |
| Ga05G0369 | cellwall | Polygalacturonase | putative polygalacturonase [Gossypium arboreum] | AT1G19170.1 | Pectin lyase-like superfamily protein |

| | | | | |
|---|---|---|---|---|
| Ga06G0835cellwall | Polygalac-turonase | putative polygalac-turonase [Gossypium arboreum] | AT3G42950.1 | Pectin lyase-like super-family protein |
| Ga06G1973cellwall | LAC5 | Laccase 12 [Theo-broma cacao] | AT2G40370.1 | laccase 5 |
| Ga11G0039cellwall | LAC4 | Laccase-17 -like pro-tein [Gossypium ar-boreum] | AT5G60020.1 | laccase 17 |
| Ga10G0166cellwall | LAC22 | Laccase-22 [Gossy-pium arboreum] | AT2G38080.1 | Laccase/Diphenol oxi-dase family protein |
| Ga09G1756cellwall | LAC17 | Laccase 17 [Theo-broma cacao] | AT5G60020.1 | laccase 17 |
| Ga11G0041cellwall | LAC17 | Laccase 17 isoform 1 [Theobroma cacao] | AT5G60020.1 | laccase 17 |
| Ga09G1729cellwall | IRX9 | putative beta-1,4-xy-losyltransferase IRX9 -like protein [Gossypium arbo-reum] | AT2G37090.1 | Nucleotide-diphospho-sugar transferases su-perfamily protein |
| Ga13G2817cellwall | IRX7 | putative glucu-ronoxylan glucu-ronosyltransferase IRX7 -like protein [Gossypium arbo-reum] | AT2G28110.1 | Exostosin family pro-tein |
| Ga01G2197cellwall | IRX12 | Laccase/Diphenol oxidase family pro-tein [Theobroma ca-cao] | AT2G38080.1 | Laccase/Diphenol oxi-dase family protein |
| Ga04G0648cellwall | IRX12 | Laccase-4 -like pro-tein [Gossypium ar-boreum] | AT2G38080.1 | Laccase/Diphenol oxi-dase family protein |
| Ga11G0024cellwall | IRX12 | Laccase/Diphenol oxidase family pro-tein isoform 1 [The-obroma cacao] | AT2G38080.1 | Laccase/Diphenol oxi-dase family protein |
| Ga03G2054cellwall | IRX10 | putative beta-1,4-xy-losyltransferase IRX10 -like protein [Gossypium arbo-reum] | AT1G27440.1 | Exostosin family pro-tein |
| Ga09G0843cellwall | CTL2 | chitinase-like protein [Gossypium hirsu-tum] | AT3G16920.1 | chitinase-like protein 2 |
| Ga10G1044cellwall | CTL2 | Chitinase-like pro-tein 2 [Gossypium arboreum] | AT3G16920.1 | chitinase-like protein 2 |
| Ga01G1918cellwall | COBL4 | COBRA-like protein 4 [Gossypium arbo-reum] | AT5G15630.1 | COBRA-like extracellu-lar glycosyl-phospha-tidyl inositol-anchored protein family |
| Ga08G0607cellwall | COBL4 | COBRA-like protein 4 [Gossypium arbo-reum] | AT5G15630.1 | COBRA-like extracellu-lar glycosyl-phospha-tidyl inositol-anchored protein family |
| Ga11G2338cellwall | COBL4 | COBRA-like protein 4 [Gossypium arbo-reum] | AT5G15630.1 | COBRA-like extracellu-lar glycosyl-phospha-tidyl inositol-anchored protein family |
| Ga10G2710cellwall | CESA8 | cellulose synthase A1 [Gossypium bar-badense] | AT4G18780.1 | cellulose synthase fam-ily protein |

| | | | | |
|---|---|---|---|---|
| Ga05G0095cellwall | CESA7 | CESA8 [Gossypium hirsutum] | AT5G17420.1 | Cellulose synthase family protein |
| Ga07G0463cellwall | CESA7 | Cellulose synthase A catalytic subunit 7 [UDP-forming] -like protein [Gossypium arboreum] | AT5G17420.1 | Cellulose synthase family protein |
| Ga07G2381cellwall | CESA4 | Cellulose synthase A catalytic subunit 4 [UDP-forming] -like protein [Gossypium arboreum] | AT5G44030.1 | cellulose synthase A4 |
| Ga08G0544cellwall | CESA4 | Cellulose synthase A catalytic subunit 3 [UDP-forming] -like protein [Gossypium arboreum] | AT5G44030.1 | cellulose synthase A4 |
| Ga07G1428cellwall | BXL2 | putative beta-D-xylosidase 2 -like protein [Gossypium arboreum] | AT1G02640.1 | beta-xylosidase 2 |
| Ga11G0347cellwall | BGLU42 | Beta-glucosidase, putative [Theobroma cacao] | AT5G36890.2 | beta glucosidase 42 |
| Ga05G2284salt | TUA | alpha-tubulin [Gossypium hirsutum] | AT1G50010.1 | tubulin alpha-2 chain |
| Ga01G1596salt | Protein kinase | hypothetical protein F383_22386 [Gossypium arboreum] | AT1G09600.1 | Protein kinase superfamily protein |
| Ga07G0340salt | PLD1 | Phospholipase D alpha 1 [Theobroma cacao] | AT3G15730.1 | phospholipase D alpha 1 |
| Ga08G0131salt | NPF2.3 | Major facilitator superfamily protein, putative [Theobroma cacao] | AT3G45680.1 | Major facilitator superfamily protein |
| Ga11G2976salt | NAC096 | NAC domain-containing 4 -like protein [Gossypium arboreum] | AT2G17040.1 | NAC domain containing protein 36 |
| Ga11G2975salt | NAC062 | NAC domain-containing 4 -like protein [Gossypium arboreum] | AT3G49530.1 | NAC domain containing protein 62 |
| Ga01G1785salt | NAC043 | NAC domain-containing protein [Gossypium hirsutum] | AT2G46770.1 | NAC (No Apical Meristem) domain transcriptional regulator superfamily protein |
| Ga10G0673salt | FLA17 | fasciclin-like arabinogalactan protein 15 [Gossypium hirsutum] | AT5G06390.1 | FASCICLIN-like arabinogalactan protein 17 precursor |
| Ga09G1563salt | CTR1 | Serine/threonine-protein kinase CTR1 [Gossypium arboreum] | AT5G03730.2 | Protein kinase superfamily protein |
| Ga11G3008salt | CRK3 | CDPK-related protein kinase [Gossypium arboreum] | AT2G46700.1 | CDPK-related kinase 3 |
| Ga08G1237salt | COMT | Caffeic acid 3-O-methyltransferase | AT5G54160.1 | O-methyltransferase 1 |

| | | | | |
|---|---|---|---|---|
| | | [Gossypium arboreum] | | |
| Ga12G0336salt | COMT | Caffeic acid 3-O-methyltransferase [Gossypium arboreum] | AT5G54160.1 | O-methyltransferase 1 |
| Ga02G0079salt | APX3 | Ascorbate peroxidase isoform 1 [Theobroma cacao] | AT4G35000.1 | ascorbate peroxidase 3 |
| Ga10G2561salt | 4CL1 | 4-coumarate--CoA ligase 1 [Gossypium arboreum] | AT1G51680.1 | 4-coumarate:CoA ligase 1 |

**Table S10.** KEGG Fst and DEG statistics.

| type | Contrastive study | carbon metabolism | genetic processing | lipid metabolism | protein processing | regulation | Secondary metabolism | other |
|---|---|---|---|---|---|---|---|---|
| DE | SC_YER | 0 | 1 | 5 | 3 | 2 | 0 | 0 |
| DE | SC_YZR | 1 | 1 | 9 | 4 | 0 | 3 | 2 |
| DE | YER_YZR | 0 | 0 | 0 | 2 | 0 | 5 | 2 |
| Fst | SC_YER | 2 | 0 | 0 | 1 | 2 | 3 | 1 |
| Fst | SC_YZR | 0 | 0 | 2 | 3 | 0 | 3 | 1 |
| Fst | YER_YZR | 0 | 1 | 2 | 0 | 0 | 5 | 0 |

**Table S11.** Statistics of duplicated gene pairs of different types.

| Type | Ka/Ks>1 | Ka/Ks1<1 | Mean of Ka/Ks |
|---|---|---|---|
| DSD | 0.03530107 | 0.96469893 | 0.4212519 |
| PD | 0.05089059 | 0.94910942 | 0.5359288 |
| TD | 0.04673457 | 0.95326543 | 0.4907648 |
| TRD | 0.01506616 | 0.98493384 | 0.3238082 |
| WGD | 0.00103665 | 0.99896335 | 0.2195169 |

**Table S12.** Primers used in this study.

| Primer name | Sequence | Purpose |
|---|---|---|
| pLL00R-proGa01G0472-HindIII-F | ACGGCCAGTGCCAAGCTTGCAG-GAAATGAAAAGGGGAA | For LUC activity assay |
| pLL00R-proGa01G0472-SalI-R | GGATCCTCTAGAGTCGACCGACATTGAC-CATCATATCG | For LUC activity assay |
